# Supplementary material for: Linking electronic mental healthcare and benefits records in South London: design, procedure and descriptive outcomes
Source: BMJ Open. 2023 Feb 14;13(2):e067136. doi: 10.1136/bmjopen-2022-067136 (PMC9950921; doi:10.1136/bmjopen-2022-067136)
Supplement: Supplementary data [file bmjopen-2022-067136supp001.pdf]

Supplementary Material

Data access

The DWP administrative data and CRIS clinical data are stored and hosted by the SLaM Clinical Data Linkage Service (CDLS). Researchers wishing to explore the DWP-CRIS data will first need to submit a project proposal to the CRIS Oversight Committee. The CRIS Oversight Committee will assess whether the application adherences to the agreed standards of research and dissemination specifically outlined for the CRIS database. Once approved, the application will be directed to the Work and Health Screening Panel, specifically set up to consider applications to explore the linked DWP-CRIS data. This panel is made up of a representative from the DWP and a member of the CRIS Oversight Committee. The decision to grant or deny approval for the application to access and use the linked data will be informed by the governance and ethical approvals obtained and implemented as part of the established linkage. These include: 1) NHS Health Research Authority Research Ethics Committee approval, 2) Section 251 approval under the NHS Health Research Authority Confidential Advisory Group, 3) SLaM Caldicott Guardian, 4) DWP governance panels and 5) DWP/CRIS data sharing agreement. In addition, all projects are required to have a local collaborator from King's Health Partners (e.g. SLaM, King's College London, King's College Hospital or Guy's and St Thomas' NHS Foundation Trust).

All approved projects are published with the proposal title, lay summary and lead researcher details on the public facing Maudsley BRC website (https://www.maudsleybrc.nihr.ac.uk/facilities/clinical-record-interactive-search-cris/cris-data-linkages). All research papers will be published in the CRIS publications section of the BRC website (https://www.maudsleybrc.nihr.ac.uk/facilities/clinical-record-interactive-search-cris/cris-publications/).

Once the Work and Health Screening Panel has approved the application, the applicant will work with the SLaM Clinical Data Linkage Service to develop a project data extraction specification, only including the data that is needed to answer the specific research questions as outlined in the project application. The analysis of specific extracts of the linked data will be carried out within the SLaM firewall by the applicant on site, or via a secure VPN connection. Only those who hold a contract with SLaM (substantive or honorary), or a research passport, will be able to submit a project application and work with the linked data once approved.

Supplementary Table 1: Overview of types of benefits received among linked patients (n=183,821).

**Type of benefit$^{\mu¬}$**	**N (%)**
Employment and Support Allowance (ESA)	82436 (44.9)
Jobseeker's Allowance (JSA)	75524 (41.1)
Income Support (IS)	59748 (32.5)
Disability Living Allowance (DLA)	52675 (28.7)
Incapacity Benefit (IB)	50520 (27.5)
Retirement / State Pension (RP)	49040 (26.7)
Personal Independence Payment (PIP)	47315 (25.7)
Universal Credit (UC)	46789 (25.4)
UC conditionality regime – Searching for work	38073 (81.4)
UC conditionality regime – Working, with requirements	13448 (28.7)
UC conditionality regime – No work requirements	16505 (35.3)
UC conditionality regime – Working, no requirements	13610 (29.1)
UC conditionality regime – Preparing for work	4497 (9.6)
UC conditionality regime – Planning for work	2402 (5.1)
Attendance Allowance (AA)	25017 (13.6)
Pension Credit (PC)	22749 (12.4)
Carer's Allowance (ICA)	13798 (7.5)
Severe Disablement Allowance (SDA)	3682 (2.0)
Passported Incapacity Benefit (PIB)	1622 (0.9)
Bereavement Benefit (BB)	732 (0.4)
Widows Benefit (WB)	326 (0.2)

µ benefit received between 1st of January 2005 and 30th of June 2020. ¬ PIP was only introduced in April 2013 to replace DLA. UC was only introduced in 2013. SDA was replaced by IB in April 2001. IB was replaced by ESA and since January 2011 no new IB claims have been accepted. % will not add up to 100% as patients could have received multiple benefits over time.

Supplementary Table 2: Overview of patients who had a recorded primary psychiatric diagnosis and had ever a benefit entry for benefits not directly related to unemployment, sickness, disability, Income Support or Universal Credit.

Benefit type / Recorded primary psychiatric diagnoses (ICD-10 code and description) ∞	Retirement / State Pension (RP) N (%) n=22605	Pension Credit (PC) N (%) n=18358	Attendance Allowance (AA) N (%) n=20870	Widow's Benefit (WB) N (%) n=224	Bereavement Benefit (BB) N (%) n=502	Carer's Allowance (ICA) N (%) n=9298	Passported Incapacity Benefit (PIB) N (%) n=1194
F00-F09 (Mental and behavioural disorders, and mental disorders due to known physiological conditions) n=26069	22605 (86.7)	9827 (37.7)	15503 (59.5)	73 (0.3)	44 (0.2)	1146 (4.4)	32 (0.1)
F10-F19 (Mental and behavioural disorders due to psychoactive substance use) n=23713	1879 (7.9)	1118 (4.7)	413 (1.7)	19 (0.1)	68 (0.3)	2002 (8.4)	89 (0.4)
F20-F29 (Schizophrenia, schizotypal, delusional disorders and other non-mood psychotic disorders) n=14944	2732 (18.3)	2042 (13.7)	715 (4.8)	19 (0.1)	39 (0.3)	520 (3.5)	183 (1.2)
F30-F39 (Mood (affective) disorders) n=27046	6502 (24.0)	2996 (11.1)	2532 (9.4)	58 (0.2)	178 (0.7)	2426 (9.0)	122 (0.5)
F40-F48 (Anxiety, dissociative, stress-related, somatoform and other nonpsychotic mental disorders) n=20432	4128 (20.2)	1765 (8.6)	1567 (7.7)	46 (0.2)	134 (0.7)	1787 (8.8)	197 (1.0)

F50-F59 (Behavioural syndromes associated with physiological disturbances and physical factors) n=3840	226 (5.9)	64 (1.7)	40 (1.0)	<5 (<1.0)	18 (0.5)	276 (7.2)	50 (1.3)
F60-F69 (Disorders of adult personality and behaviour) n=5495	316 (5.8)	205 (3.7)	64 (1.2)	<5 (<1.0)	12 (0.2)	437 (8.0)	77 (1.4)
F70-F79 (Intellectual disabilities) n=2448	233 (9.5)	299 (12.2)	26 (1.1)	<5 (<1.0)	<5 (<1.0)	41 (1.7)	232 (9.5)
F80-F89 (Pervasive and specific developmental disorders) n=2623	39 (1.5)	20 (0.8)	5 (0.2)	<5 (<1.0)	<5 (<1.0)	145 (5.5)	116 (4.4)
F90-F98 (Behavioural and emotional disorders with onset usually occurring in childhood and adolescence) n-5092	59 (1.2)	22 (0.4)	5 (0.1)	<5(<1.0)	6 (0.1)	518 (10.2)	96 (1.9)

∞ latest psychiatric primary diagnosis recorded closest and before window end date (30 June 2019) based on ICD-10 F codes only (mental and behavioural disorders) but excluding non-specific diagnoses, e.g. Z, F99*, FXX. µ any type of benefits received between 1st of January 2005 and 30th of June 2020.* Cell sizes with less than <5 observations are shown as <5 (<1.0%).

Supplementary Table 3: Overview of patients who had a recorded primary psychiatric diagnosis and had received Universal Credit, by Universal Credit conditionality regime.

**Benefit type** / **Recorded primary psychiatric diagnoses (ICD-10 code and description) ∞**	**UC Conditionality regime – searching for work (AA)** **N (%)** n=25012	**UC Conditionality regime – working, with requirements (AB)** **N (%)** n=8409	**UC Conditionality regime – no work requirements (BC)** **N (%)** n=11404	**UC Conditionality regime – working, no requirements (BD)** **N (%)** n=8450	**UC Conditionality regime – preparing for work (CE)** **N (%)** n=2991	**UC Conditionality regime – planning for work (DF)** **N (%)** n=1488
F00-F09 (Mental and behavioural disorders, and mental disorders due to known physiological conditions) n=513	415 (80.9)	129 (25.2)	240 (46.8)	117 (22.8)	36 (7.0)	6 (1.2)
F10-F19 (Mental and behavioural disorders due to psychoactive substance use) n=8547	7605 (88.7)	1911 (22.3)	2524 (29.4)	1809 (21.1)	807 (9.4)	185 (2.2)
F20-F29 (Schizophrenia, schizotypal, delusional disorders and other non-mood psychotic disorders) n=2989	2467 (85.1)	762 (26.3)	1427 (49.2)	638 (22.0)	113 (3.9)	52 (1.8)
F30-F39 (Mood (affective) disorders) n=7044	5437 (77.2)	2212 (31.4)	2814 (40.0)	2322 (33.0)	866 (12.3)	553 (7.9)
F40-F48 (Anxiety, dissociative, stress-related, somatoform and	4197 (77.0)	1744 (32.0)	2003 (36.8)	1805 (33.1)	650 (11.9)	364 (6.7)

other nonpsychotic mental disorders) n=5451						
F50-F59 (Behavioural syndromes associated with physiological disturbances and physical factors) n=1168	831 (71.2)	332 (28.4)	346 (29.6)	484 (41.4)	110 (9.4)	95 (8.1)
F60-F69 (Disorders of adult personality and behaviour) n=1874	1500 (80.0)	448 (26.0)	934 (49.8)	494 (26.4)	180 (9.6)	94 (5.0)
F70-F79 (Intellectual disabilities) n=238	195 (81.9)	32 (13.5)	143 (60.1)	18 (7.6)	20 (8.4)	5 (2.1)
F80-F89 (Pervasive and specific developmental disorders) n=653	551 (84.4)	158 (24.2)	285 (43.6)	111 (17.0)	53 (8.1)	17 (2.6)
F90-F98 (Behavioural and emotional disorders with onset usually occurring in childhood and adolescence) n=2209	1814 (82.1)	641 (29.0)	688 (31.2)	652 (29.5)	156 (7.1)	117 (5.3)

∞ latest psychiatric primary diagnosis recorded closest and before window end date (30 June 2019) based on ICD 10 F codes only (mental and behavioural disorders) but excluding non-specific diagnoses, e.g. Z, F99*, FXX. µ any type of benefits received between 1st of January 2005 and 30th of June 2020.*
