# Supplementary material for: Linking electronic mental healthcare and benefits records in South London: design, procedure and descriptive outcomes
Source: BMJ Open. 2023 Feb 14;13(2):e067136. doi: 10.1136/bmjopen-2022-067136 (PMC9950921; doi:10.1136/bmjopen-2022-067136)
Supplement: Author's manuscript [file bmjopen-2022-067136.draft_revisions.pdf]

BMJ Open

BMJ Open is committed to open peer review. As part of this commitment we make the peer review history of every article we publish publicly available.

When an article is published we post the peer reviewers' comments and the authors' responses online. We also post the versions of the paper that were used during peer review. These are the versions that the peer review comments apply to.

The versions of the paper that follow are the versions that were submitted during the peer review process. They are not the versions of record or the final published versions. They should not be cited or distributed as the published version of this manuscript.

BMJ Open is an open access journal and the full, final, typeset and author-corrected version of record of the manuscript is available on our site with no access controls, subscription charges or pay-per-view fees (http://bmjopen.bmj.com).

If you have any questions on BMJ Open's open peer review process please email info.bmjopen@bmj.com

BMJ Open

Linking electronic mental healthcare and benefits records in South London: design, procedure, and descriptive outcomes.

Journal:	*BMJ Open*
Manuscript ID	bmjopen-2022-067136
Article Type:	Original research
Date Submitted by the Author:	03-Aug-2022
Complete List of Authors:	Stevelink, Sharon; King's College London Institute of Psychiatry Psychology and Neuroscience, Department of Psychological Medicine; King's College London Institute of Psychiatry Psychology and Neuroscience, King's Centre for Military Health Research, Department of Psychological Medicine<br>Phillips, Ava; King's College London Institute of Psychiatry Psychology and Neuroscience, Department of Psychological Medicine<br>Broadbent, Matthew; NIHR Maudsley Biomedical Research Centre, South London and Maudsley Mental Health NHS Trust<br>Boyd, Andy; University of Bristol Medical School, Population Health Sciences<br>Dorrington, Sarah; King's College London Institute of Psychiatry Psychology and Neuroscience; NIHR Maudsley Biomedical Research Centre, South London and Maudsley Mental Health NHS Trust<br>Jewell, Amelia; NIHR Maudsley Biomedical Research Centre, South London and Maudsley Mental Health NHS Trust<br>Leal, Ray; King's College London Institute of Psychiatry Psychology and Neuroscience, King's Centre for Military Health Research, Department of Psychological Medicine; King's College London Institute of Psychiatry Psychology and Neuroscience, Department of Psychological Medicine<br>Bakolis, Ioannis; King's College London Institute of Psychiatry Psychology and Neuroscience, Health Services and Population Research Department; King's College London Institute of Psychiatry Psychology and Neuroscience, Department of Biostatistics and Health Informatics<br>MADAN, IRA; Guy's and St Thomas' Hospitals NHS Trust, Department of Occupational Health; King's College London<br>Hotopf, Matthew; King's College London Institute of Psychiatry Psychology and Neuroscience, Department of Psychological Medicine; NIHR Maudsley Biomedical Research Centre, South London and Maudsley Mental Health NHS Trust<br>Fear, Nicola; King's College London Institute of Psychiatry Psychology and Neuroscience, King's Centre for Military Health Research, Department of Psychological Medicine; King's College London Institute of Psychiatry Psychology and Neuroscience, Academic Department of Military Mental Health, Department of Psychological Medicine<br>Downs, Johnny; King's College London Institute of Psychiatry Psychology and Neuroscience, Department of Psychological Medicine; NIHR Maudsley Biomedical Research Centre, South London and Maudsley Mental Health NHS Trust
Keywords:	Epidemiology < TROPICAL MEDICINE, MENTAL HEALTH, Public health <

	INFECTIOUS DISEASES

For peer review only - http://bmjopen.bmj.com/site/about/guidelines.xhtml

I, the Submitting Author has the right to grant and does grant on behalf of all authors of the Work (as defined in the below author licence), an exclusive licence and/or a non-exclusive licence for contributions from authors who are: i) UK Crown employees; ii) where BMJ has agreed a CC-BY licence shall apply, and/or iii) in accordance with the terms applicable for US Federal Government officers or employees acting as part of their official duties; on a worldwide, perpetual, irrevocable, royalty-free basis to BMJ Publishing Group Ltd ("BMJ") its licensees and where the relevant Journal is co-owned by BMJ to the co-owners of the Journal, to publish the Work in this journal and any other BMJ products and to exploit all rights, as set out in our licence.

The Submitting Author accepts and understands that any supply made under these terms is made by BMJ to the Submitting Author unless you are acting as an employee on behalf of your employer or a postgraduate student of an affiliated institution which is paying any applicable article publishing charge ("APC") for Open Access articles. Where the Submitting Author wishes to make the Work available on an Open Access basis (and intends to pay the relevant APC), the terms of reuse of such Open Access shall be governed by a Creative Commons licence – details of these licences and which Creative Commons licence will apply to this Work are set out in our licence referred to above.

Other than as permitted in any relevant BMJ Author's Self Archiving Policies, I confirm this Work has not been accepted for publication elsewhere, is not being considered for publication elsewhere and does not duplicate material already published. I confirm all authors consent to publication of this Work and authorise the granting of this licence.

For peer review only

Linking electronic mental healthcare and benefits records in South London: design, procedure, and descriptive outcomes.

Sharon A.M. Stevelink# [1,2], Ava Phillips# [1], Matthew Broadbent [3], Andy Boyd [4], Sarah Dorrington [1,3], Amelia Jewell [3], Ray Leal [1,2], Ioannis Bakolis [5,6], Ira Madan [7], Matthew Hotopf [1,3], Nicola T. Fear* [1,2,8], Johnny Downs* [1,3].

# joint first authors

* Joint last authors

1. Department of Psychological Medicine, Institute of Psychiatry, Psychology and Neuroscience, King's College London, London, UK.
2. King's Centre for Military Health Research, Department of Psychological Medicine, Institute of Psychiatry, Psychology and Neuroscience, King's College London, London, UK.
3. NIHR Maudsley Biomedical Research Centre, South London and Maudsley Mental Health NHS Trust, London, UK.
4. Population Health Sciences, Bristol Medical School, University of Bristol, Bristol, UK.
5. Centre for Implementation Science, Health Services and Population Research Department, Institute of Psychiatry, Psychology and Neuroscience, London, UK.
6. Department of Biostatistics and Health Informatics, Institute of Psychiatry, Psychology and Neuroscience, London, United Kingdom.
7. Department of Occupational Health, Guy's and St Thomas' Hospitals NHS Trust, London, UK and King's College London, London, UK.
8. Academic Department of Military Mental Health, Department of Psychological Medicine, Institute of Psychiatry, Psychology and Neuroscience, King's College London, London, UK.

Corresponding author: Dr Sharon Stevelink, Institute of Psychiatry, Psychology and Neuroscience, King's College London, 16 De Crespigny Park, London, UK, SE5 9AF.

Email: Sharon.stevelink@kcl.ac.uk

Word count: 4722

Key words: benefits, data linkage, Department for Work and Pensions, epidemiology, electronic healthcare records, mental health, public health, South London and Maudsley NHS Foundation Trust, welfare state.

For peer review only

Abstract

Objectives: To describe the process and outcomes of a novel data linkage between electronic secondary mental healthcare records from the South London and Maudsley (SLaM) NHS Foundation Trust with benefits records from the Department for Work and Pensions (DWP). We also describe the mental health and benefit profile of patients who were successfully linked.

Design: A deterministic linkage of routine records from UK health and welfare government service providers within a secure research environment.

Setting and participants: Adults aged ≥18 years who were referred to or accessed treatment at SLaM services between January 2007 and June 2019, including those who were treated as part of the Improving Access to Psychological Therapies (IAPT) services between January 2008 and June 2019 (n=448,404). Benefits data from the DWP from January 2005 until June 2020.

Outcome measures: The linkage rate and associated socio-demographic, diagnostic and treatment factors. Recorded primary psychiatric diagnosis based on International Classification of Diseases (ICD) 10 codes and type of benefit receipt.

Results: A linkage rate of 92.3% was achieved. Women, younger patients, and those from ethnic minority groups were less likely to be successfully linked. Patients who had died, had a recorded primary psychiatric diagnosis, had also engaged with IAPT services, and had a higher number of historical postcodes available were more likely to be successfully linked. Eighty-three percent of patients received benefits. Benefit receipt across the psychiatric diagnosis spectrum was high, over 80% across most ICD-10 codes.

Conclusions: This data linkage is the first of its kind to demonstrate the use of routinely collected mental health and benefits data. Benefit receipt was high among patients accessing secondary mental healthcare services and varied by psychiatric diagnosis. Future areas of research are discussed, including exploring the effectiveness of interventions for helping people into work, and the impact of benefit reforms.

For peer review only

Summary

- This is a novel data linkage between electronic mental healthcare records and benefits records providing the opportunity to answer important questions relating to mental health, work, and benefit receipt.
- A high linkage rate of 92.3% was achieved.
- The sample does not include a comparison group (e.g., people who did not access secondary mental healthcare services).
- Although there are indicators of people being in and out of work depending on what type of benefits are being received (unemployment related benefits), there is no reliable employment variable within the data stating whether someone is currently in or out of work (except for Universal Credit).
- There is a potential for linkage bias as a result of the method used (ad hoc deterministic fuzzy matching) and having no unique identifier between data sets.

For peer review only

Introduction

In the UK, approximately 1.8 million people face long-term sickness absence of four weeks or longer, costing our society £100 billion annually (1). Long-term sickness absence is associated with social exclusion, poor health outcomes and high mortality (2-4). Each year, over 300,000 people are forced to leave work due to health problems (5). Mental disorders are one of the most common causes of sickness absence and subsequent long-term occupational disability (6, 7). In 2019/2020, 17.9 million working days were lost due to mental ill health (8). For many who access mental health services, their difficulties impact on their ability to work. Understanding people's finances, welfare, benefits, and occupational needs are integral to the care and quality of life for people with mental disorders, however these are often overlooked.

Over the last decade, major changes have taken place in the UK benefits system including the extension of benefit sanctions (9); the introduction of 'Universal Credit' (UC), a means-tested benefit replacing six benefits and tax credits for those of working-age (10); the introduction of work capability assessments (WCA) where one's capability for work-related activity is reviewed; and an increased reliance on conditionality meaning that people need to fulfil certain work-related activity requirements to maintain their full benefit entitlements. These were announced as part of the *Welfare Reform Act 2007* and *2012*, and *Welfare Reform and Work Act 2016*. These changes have been met with concern about their impact on people's well-being, and particularly on those with mental disorders (11-16). Hence, research into the welfare and benefit needs of the population with mental disorders is required, to inform policy on welfare provision when this group is at their most vulnerable; also to support return to work as an integral part of recovery for people who are able to return to employment (17, 18). The latter is especially relevant given the introduction of, for example, Improving Access to Psychological Therapies services (19) and Individual Placement and Support Services (20) in the UK.

There are no pre-existing datasets that can currently address this. Alone, NHS healthcare records are an unreliable source of information on benefit receipts or employment status; these are not routinely collected or recorded. Data held by the Department for Work and Pensions (DWP) which records national welfare and public service interactions in the UK, for example on unemployment-related benefits, is devoid of high-quality information about health status. The limited data that is available in these benefits records are solely based on diagnostic information provided in benefit applications for specific benefits, and these are often incomplete

The advent of electronic healthcare records and systems, and the increasing sophistication with which data can be linked and analysed, has presented the opportunity to change the research landscape. We report here on a unique linkage of welfare and benefits data with routinely collected mental health data of over 400,000 adults referred to psychiatric services, enabling us to address gaps in evidence regarding the interrelationships between benefit receipt, employment status, mental disorders, treatment, well-being and recovery. To our knowledge, this is the first time in the UK that routine health records have been linked with benefits data.

Here, we describe the process and outcomes of linking electronic mental healthcare records from patients who accessed secondary mental healthcare services at the South London and Maudsley

For peer review only

(SLaM) NHS Foundation Trust with benefits records from the DWP. First, we will describe the ethical and governance considerations encountered before we could proceed with the linkage. Second, we describe the approach, data linkage rate and factors associated with successful linkage. Finally, we provide an overview of the mental health and benefit profile of patients who were successfully linked.

Methods

Data sources

South London and Maudsley NHS Foundation Trust Biomedical Research Centre Case Register

The SLaM NHS Foundation Trust is one of Europe's largest providers of secondary mental healthcare services, providing care predominantly for the South London boroughs of Lambeth, Lewisham, Southwark, and Croydon, covering a catchment area of over 1.2 million residents. SLaM provides specialist (secondary) mental healthcare services as well as IAPT services. The SLaM Biomedical Research Centre (BRC) Case Register includes electronic mental healthcare records of patients accessing SLaM. In 2008, the Clinical Records Interactive Search (CRIS) system was developed (21) to curate deidentified data from SLaM's electronic mental healthcare records for research use. Information concerning patients' mental healthcare journey is available in pseudo-anonymised format either in free clinical text notes or structured fields as part of a patient's electronic mental healthcare record. CRIS clinical data may include, for example, individual level data on socio-demographic characteristics (e.g. month and year of birth, sex, ethnicity, neighbourhood deprivation), time variant data on International Classification of Diseases (ICD)-10 psychiatric diagnosis, diagnostic assessments, mental health treatment (e.g. local or specialist services, community vs. inpatient), service use (e.g. patterns of engagement), medication prescriptions and psychotherapeutic interventions. CRIS data covered the 1st of January 2007 till the 30th of June 2019.

Department for Work and Pensions benefits data

The DWP in the UK is responsible for the implementation of policy regarding welfare and state benefits. Benefits data includes individual level demographic data (e.g. date of death, and sex), time variant data related to the on and off flows of benefits (e.g. Incapacity Benefit, Carers Allowance, Income Support, Jobseekers Allowance, Attendance Allowance, Retirement/State Pension, Disability Living Allowance, Severe Disablement Benefit, Widows Benefit, Pension Credit, Passported Incapacity Benefit, Bereavement Benefit, Employment Support, Universal Credit, Personal Independence Payment and relevant benefit specific details) (22). Start and end dates of benefit spells are provided as well as the amount of money received. In addition, information is provided about WCA and work programme access. Benefits data covered 1st of January 2005 till 30th of June 2020.

Sample

The sample consists of all adults (aged 18 years and older) who 1) have been referred for treatment with SLaM secondary mental healthcare services between 1st January 2007 (the implementation of electronic mental healthcare records across SLaM secondary mental healthcare services was only finalised by that time) to 30th June 2019, or 2) had an event with SLaM secondary mental healthcare services during this time period and were aged 18 or over at the time of their latest recorded event in the window, or 3) patients who had a treatment episode at the Improving Access to Psychological Therapies (IAPT) services between 1st January 2008 to 30th June 2019 were included. Patients ranged

For peer review only - http://bmjopen.bmj.com/site/about/guidelines.xhtml

in symptom severity from common mental disorders to serious mental illness (e.g. schizophrenia, bipolar affective disorder), substance use disorders and organic disorders (e.g. neurological syndromes associated with severe intellectual impairment). For the current paper, we only focused on the linkage of patients who accessed specialist (secondary) mental health care services within SLaM (and possibly also IAPT) but not those who only accessed IAPT services within SLaM. This decision was made as we were especially interested in the former group of patients who were more likely to have severe mental health symptomatology.

Patient and public involvement and engagement

The proposed linkage of electronic mental healthcare records of SLaM and benefits records from the DWP was presented to the Maudsley Biomedical Research Centre Data Linkage Service User and Carer Advisory Group in December 2016 (23). The members of the Advisory Group experienced mental ill health themselves or as a carer for someone with a mental health diagnosis and were accessing or had accessed mental healthcare services. All were given training concerning data linkages, the underlying clinical research information system, data security, governance, and the research environment at SLaM.

The members of the Advisory Group were supportive of the proposed linkage when first discussed in December 2016. The linkage was presented again in September 2019 with a discussion around the specific research questions and opportunities for continued patient and public involvement in the project. They will be consulted on a regular basis now the data linkage has been finalised with a focus on discussing preliminary results and gathering input regarding dissemination and impact strategies.

Ethical and governance approvals

We submitted the proposed linkage to the South Central – Oxford C Research Ethics Committee for ethical approval. A favourable opinion was received in 2017 (ref 17/SC/0581). In addition, we successfully applied in 2017 for Section 251 approval under the NHS Health Research Authority Confidential Advisory Group (ref 17CAG0055). We believed that it was not practical or appropriate for the proposed linkage to be successfully achieved through a consent-based methodology.

Once ethical approvals were in place, we developed a data sharing agreement. This agreement outlines the data sharing agreements between SLaM and the Secretary of State for Work and Pensions in relation to the data linkage. The agreement sets out lawful basis of the data linkage as well as the principles and procedures for data sharing and the use of the linked data. Details on how to access the linked data can be found in the Supplementary Material (Supplement 1).

Data linkage process

The linkage of CRIS clinical records with benefits data took place in late 2020. An ad hoc deterministic matching approach was used, namely fuzzy matching, based on personal identifiers held on the DWP's Customer Information System (CIS) which hosts a 'spine' record of everyone who has ever been issued a National Insurance Number (NINO). The NINO is a unique individual ID allocated for employment, tax, and welfare purposes.

1. The SLaM Clinical Data Linkage Service, 'a trusted third party', shared the personal identifiers of the eligible sample (patient name, date of birth, sex, postcode and postcode history) and

For peer review only - http://bmjopen.bmj.com/site/about/guidelines.xhtml

the BRCID pseudonym used within the CRIS database with DWP (the data were transferred using the secure 'Egress' system).

2. The DWP linked the SLaM personal identifiers to DWP held personal identifiers in a secure area using a fuzzy-matching process (uniqueness cut-off threshold of 90% or above) to create a table linking the BRCID pseudonym to a NINO (where possible). Approved benefits data were extracted from DWP systems using the NINO.
3. The NINO was replaced with the BRCID pseudonym before the linked de-identified DWP benefits data were sent back to the SLaM Clinical Data Linkage Service via Egress. At no point were SLaM clinical data shared. DWP destroyed the SLaM personal identifiers once the matching work was complete.
4. The benefits data with the attached BRCIDs are stored within the SLaM secure research system in a separate database to the CRIS clinical data with access to restricted users only.
5. The benefits data and CRIS clinical data are only joined on a project specific basis, after the necessary approvals have been given. BRCIDs are stripped before a project specific anonymised data set is provided to the researcher.

Materials

The following socio-demographic and clinical, diagnostic and treatment variables were derived from the linked data for further exploration. These were selected based on data availability, previous research indicating that these factors were found to be associated with data linkage success (24, 25), and discussions within the wider research team.

Socio-demographic variables

All socio-demographic variables were derived from the clinical data, except for patient sex (male/female) as this was more complete in the benefits data. However, if sex was missing in the benefits data, and available in the clinical data, this was backfilled accordingly. Age was calculated using month and year of birth until the SLaM window end date (30th June 2019). Subsequently, age was grouped in the following categories: ≤20, 21-40, 41-60 and >60. Ethnicity was categorised as follows: White /Black, African, Caribbean, Black British/ Asian, Asian British/Mixed, Multiple racial and ethnic groups/ Other racial and ethnic minority groups and 'not stated'. We also had information on whether people had died (month and year) that resulted in a binary death (yes/no) variable. The Index of Multiple Deprivation (IMD) was informed by 2019 data, and we used the postcode closest to and before the SLaM window end date to inform IMD quintiles, with the first quintile indicating most deprived and fifth quintile least deprived. IMD is a summary measure of relative deprivation informed by 7 domains, namely income, employment, education, crime, housing, health and living environment at lower levels of geography (26). We created a variable indicating whether patients lived in the local catchment area based on Lower-layer Super Output Areas (LSOA11), a small geographical area covering a similar population size, again using the postcode closest to and before the SLaM window end date (26). In addition, we generated a categorical variable indicating the number of historical postcodes sent to DWP to facilitate the linkage for each patient (up to five maximum).

Diagnostic and treatment variables

We created a binary primary psychiatric diagnosis variable (yes/no) that referred to whether a psychiatric primary diagnosis was recorded in a patient's record closest and before the SLaM window

For peer review only

end date (30th June 2019). This only included the ICD 10 'F codes' referring to mental and behavioural disorders, thereby excluding non-specific diagnoses (e.g. Z*, F99*, FXX). Subsequently, we derived a variable outlining the type of diagnosis code patients were given, if any (ranging from F00-F09 (Mental and behavioural disorders, and mental disorders due to known physiological conditions) to F90-F98 (Behavioural and emotional disorders with onset usually occurring in childhood and adolescence). We also explored whether patients had accessed IAPT (yes/no), in addition to SLaM services between 2008 and 30th June 2019. IAPT was only introduced in 2008 so this was the earliest possible start date. Two binary variables were created (before and after 2010) to indicate patients' first and last contact with SLaM. Age at first presentation to SLaM (≤20, 21-40, 41-60, >60) was calculated using month and year of birth and the patients' earliest accepted referral date to SLaM closest to and before the SLaM window end date.

Benefit variables

Participants who were successfully linked to a NINO and had received one of the following benefits between 1st of January 2005 till 30th of June 2020 were identified as benefit recipients: Employment Support Allowance (ESA), Job Seekers Allowance (JSA), Income Support (IS), Disability Living Allowance (DLA), Incapacity Benefit (IB), Retirement/State Pension (RP), Personal Independence Pay (PIP), Universal Credit (UC), Pension Credit (PC), Carer's Allowance (ICA), Severe Disablement Allowance (SDA), Passported Incapacity Benefit (PIB) or Windows Benefit (WB) (22). We also had information on what UC conditionality regime patients were allocated to namely 1) searching for work, 2) working, with requirements, 3) no work requirements 4) working, no requirements, 5) preparing for work, or 6) planning for work (27).

Statistical analysis

Analysis of linkage bias

All statistical analyses were performed using the statistical package STATA (version 15). All variables were checked for completeness and outliers. Variable completeness and accuracy were improved by backfilling data (using the clinical or benefits records were possible). If outliers were identified, for example date of birth (as based on the age inclusion criteria), this was recoded as missing (n=14). The same was done for negative values (e.g., age at first contact n=192) and improbable dates (e.g., having accessed SLaM before it was established n=2210).

The overall linkage rate was determined by calculating the proportion of unique BRCIDs successfully linked to a NINO. We did not expect all patients to have engaged with the DWP to apply for benefits or subsequently successfully received benefits. For example, some participants engaged with the DWP, and a note was made on their benefits record, but they did not meet the criteria to claim, for example, Employment Support Allowance. Therefore, of those successfully linked to a NINO, we also calculated the proportion who had engaged with the DWP, as well as the proportion who had engaged and successfully applied for benefits according to the benefits records.

We then conducted univariable logistic regression analysis to explore socio-demographic, diagnostic and treatment related factors, associated with linkage to benefits records. We also conducted multivariable analyses thereby adjusting for factors identified *a priori* (namely age, sex and ethnicity) (24, 25). Subsequently, we generated a probability estimate of matching as a function of the risk variables with the use of the logistic regression model.

For peer review only

Sample profile

Multivariable logistic regression models were also employed to explore factors associated with benefit receipt, adjusting for age, sex and ethnicity. In addition, descriptive statistics were used to describe the benefit and the mental health profile of successfully linked patients. The latter was based on the most recently recorded ICD-10 primary psychiatric diagnostic code. We also tabulated the mental health profile of our sample by type of benefit receipt. Odds Ratios (OR), Adjusted Odds Ratios (AOR), 95% Confidence Intervals (CI) and p-values are reported.

Results

Overview of data linkage process and analysis of linkage bias

Unique IDs of 448,404 patients who accessed SLaM services (specialist (secondary) mental healthcare services and/or IAPT) were sent to the DWP (Figure 1). For this study, we only report on patients who accessed secondary mental healthcare services at SLaM (n=239,714). Of these, 221,243 (92.3%) were successfully linked to a NINO held by the DWP. Individuals identified as being under the age of 16 according to the personal details held by the DWP and those who resided in Northern Ireland at some point during benefit receipt were excluded from the data sent back to the SLaM Clinical Data Linkage Service, resulting in 220,332 (91.9%) unique linked IDs available for research purposes.

For peer review only

Results from adjusted logistic regression analyses indicated that the following groups of patients were less likely to be linked (an OR greater than 1 denotes greater chance of successful linkage compared with the reference): female patients vs. male patients, ethnic minority groups vs. patients from a white ethnic background, and middle-aged patients vs. younger patients (<21 years) (Table 1). Further, the linkage rate was also higher among patients who had a higher number of historical postcodes available. On the other hand, older patients (>60 years) were more likely to be linked than younger patients. We also found that those who had died, had a recorded psychiatric primary diagnosis, had engaged with IAPT services and accessed SLaM services more recently were more likely to be successfully linked (Table 2).

Socio-demographic, diagnostic and treatment related factors associated with benefit receipt

Of the patients who were successfully linked, 184,152 (83.6%) had engaged with the DWP, meaning they had a benefits record but not necessarily successfully claimed benefits. Among the successfully linked patients who had engaged, 183,821 (99.8%) had received benefits at some point between the 1st January 2005 and 30th June 2020 (Table 3). Adjusted results indicated that benefit receipt was higher among men, those over the age of 20 years compared with younger patients, those who had died, had a recorded primary psychiatric diagnosis and patients living in an area of higher deprivation. Patients from a black ethnic group and those from a mixed ethnic group were more likely to report benefit receipt compared to patients from other ethnic backgrounds.

For peer review only

Table 1: Comparison of socio-demographic characteristics of linked and unlinked patients with benefits data (n=239,714).

	Total N (%)	Linked N (%)	Non-linked N (%)	OR (95% CI) for successful linkage	p-value	AOR# (95% CI) for successful linkage	p-value
**Overall**	239714 (100.0)	221243 (100.0)	18471 (100.0)	-			
**Sex$**	239690 (100.0)						
Male		109321 (49.4)	8215 (44.5)	Reference		Reference	
Female		111921 (50.6)	10233 (55.5)	0.82 (0.80 to 0.85)*	p<0.001	0.81 (0.79 to 0.84)*	p<0.001
**Age (years)¥**	239699 (100.0)						
≤20		2502 (1.1)	142 (0.8)	Reference		Reference	
21-40		77943 (35.2)	9033 (48.9)	0.49 (0.41 to 0.58)*	p<0.001	0.49 (0.42 to 0.59)*	p<0.001
41-60		75860 (34.3)	6839 (37.0)	0.63 (0.53 to 0.75)*	p<0.001	0.62 (0.52 to 0.73)*	p<0.001
>60		64935 (29.4)	2445 (13.3)	1.51 (1.27 to 1.79)*	p<0.001	1.40 (1.17 to 1.66)*	p<0.001
**Ethnicity**	239714 (100.0)						
White		125244 (56.6)	7405 (40.1)	Reference		Reference	
Black/African/Caribbean/Black British		30464 (13.8)	3495 (18.9)	0.52 (0.49 to 0.54)*	p<0.001	0.56 (0.53 to 0.58)*	p<0.001
Asian/Asian British		10812 (4.9)	1708 (9.3)	0.37 (0.35 to 0.40)*	p<0.001	0.40 (0.38 to 0.42)*	p<0.001
Mixed/Multiple racial and ethnic groups		4225 (1.9)	346 (1.9)	0.72 (0.65 to 0.81)*	p<0.001	0.93 (0.83 to 1.04)*	p=0.177
Other racial and ethnic minority groups		12099 (5.5)	1889 (10.2)	0.38 (0.36 to 0.40)*	p<0.001	0.44 (0.42 to 0.46)*	p<0.001
Not stated˜		38399 (17.4)	3628 (19.6)	0.63 (0.60 to 0.65)*	p<0.001	0.74 (0.71 to 0.78)*	p<0.001
**Death^**	239714 (100.0)						
No		174820 (79.0)	17063 (92.4)	Reference		Reference	

For peer review only

Yes		46423 (21.0)	1408 (7.6)	3.22 (3.04 to 3.40)*	p<0.001	1.91 (1.79 to 2.03)*	p<0.001
**Deprivation (IMD quintile)≠**	227755 (95.0)						
First (most deprived)		46403 (21.9)	3390 (21.6)	Reference		Reference	
Second		81207 (38.3)	6536 (41.7)	0.91 (0.87 to 0.95)*	p<0.001	0.90 (0.86 to 0.94)*	p<0.001
Third		46443 (21.9)	3546 (22.6)	0.96 (0.91 to 1.00)	p=0.076	0.92 (0.87 to 0.96)*	p=0.001
Fourth		23774 (11.2)	1430 (9.1)	1.21 (1.14 to 1.29)*	p<0.001	1.09 (1.02 to 1.19)*	P=0.012
Fifth (least deprived)		14165 (6.7)	779 (5.0)	1.33 (1.23 to 1.44)*	p<0.001	1.14 (1.05 to 1.24)*	P=0.001
**Resident within local catchment area$^{\pi}$**	227997 (95.0)						
Yes		146860 (69.2)	11177 (71.2)	1.06 (1.02 to 1.11)*	p<0.001	1.03 (0.99 to 1.08)*	p<0.001
No		65435 (30.8)	4525 (28.8)	Reference		Reference	
**Number of home/residential postcodes available**	236412 (98.6)						
1		118603 (54.2)	10374 (59.6)	Reference		Reference	
2		47538 (21.7)	3474 (20.0)	1.20 (1.15 to 1.25)*	p<0.001	1.23 (1.19 to 1.29)*	p<0.001
3		22252 (10.2)	1497 (8.6)	1.30 (1.23 to 1.38)*	p<0.001	1.39 (1.32 to 1.48)*	p<0.001
4		11733 (5.4)	813 (4.7)	1.26 (1.17 to 1.36)*	p<0.001	1.41 (1.31 to 1.52)*	p<0.001
5		18885 (8.6)	1243 (7.1)	1.33 (1.25 to 1.41)*	p<0.001	1.57 (1.47 to 1.67)*	p<0.001

** P-value ≤0.01; AOR: Adjusted Odds Ratio; CI: Confidence Interval; IMD: Index of Multiple Deprivation; OR: Odds Ratio; $ based on DWP data, but if missing backfilled with SLaM data ¥ at window end date (30 June 2019), based on CRIS data; ~ includes not known, not stated or missing; ≠ IMD scores published in 2019, postcode used closest and before window end date (30 June 2019); π based on Lower-layer Super Output Areas (LSOA11) informed by postcode details closest to and before window end date (30 June 2019); ^ based on CRIS data, but if a death was recorded in benefits data but not recorded in CRIS data it was backfilled accordingly; #AOR: adjusted for age (continuous), sex and ethnicity.*

For peer review only

Table 2: Comparison of diagnostic and treatment characteristics of linked and unlinked patients with benefits data (n=239,714).

	Total N (%)	Linked N (%)	Non-linked N (%)	OR (95% CI) for successful linkage	p-value	AOR# (95% CI) for successful linkage	p-value
**Overall**	239714 (100.0)	221243 (100.0)	18471 (100.0)	-			
**Primary psychiatric diagnosis recorded∞**	239714 (100.0)						
Yes		154354 (69.8)	10997 (59.5)	1.57 (1.52 to 1.62)*	p<0.001	1.43 (1.38 to 1.48)*	p<0.001
No		66889 (30.2)	7474 (40.5)	Reference		Reference	p<0.001
**Accessed IAPTΩ**	239714 (100.0)						
Yes		50899 (23.0)	3381 (18.3)	1.33 (1.28 to 1.39)*	p<0.001	1.69 (1.63 to 1.76)*	p<0.001
No		170344 (77.0)	15090 (81.7)	Reference		Reference	
**First contact with SLaM**	233186 (97.3)						
Before 2010		80388 (37.3)	7232 (40.4)	Reference		Reference	
After 2010		134887 (62.7)	10679 (59.6)	1.14 (1.10 to 1.17)*	p<0.001	1.32 (1.28 to 1.37)*	p<0.001
**Last contact with SLaM**	235396 (98.4)						
Before 2010		36078 (16.6)	4546 (25.3)	Reference		Reference	
After 2010		181341 (83.4)	13431 (74.7)	1.70 (1.64 to 1.76)*	p<0.001	2.08 (2.01 to 2.16)*	p<0.001
**Age (years) at first presentation to SLaM**	235204 (98.1)						
≤20		23926 (11.0)	2106 (11.7)	Reference		Reference	
21-40		92178 (42.4)	10834 (60.3)	0.75 (0.71 to 0.79)*	p<0.001	0.67 (0.63 to 0.71)*	p<0.001
41-60		55388 (25.5)	3593 (20.0)	1.36 (1.28 to 1.43)*	p<0.001	0.98 (0.90 to 1.07)	p=0.637
>60		45754 (21.1)	1427 (8.0)	2.82 (2.63 to 3.02)*	p<0.001	1.53 (1.33 to 1.76)*	p<0.001

For peer review only

** P-value ≤0.01; AOR: Adjusted Odds Ratio; CI: Confidence Interval; IAPT: Improving Access to Psychological Therapies; OR: Odds Ratio; ∞ latest psychiatric primary diagnosis recorded closest and before window end date (30 June 2019) based on ICD-10 F codes only (mental and behavioural disorders) but excluding non-specific diagnoses, e.g. Z*, F99*, FXX. Ω Accessed IAPT between 2008 and 30 June 2019. #AOR: adjusted for age (continuous), sex and ethnicity.*

For peer review only

Table 3: Overview of characteristics of those who did or did not ever receive any benefits among linked patients (n=220,332).

	Total N (%)	Never received benefits$^{\Sigma}$ N (%)	Ever received benefits N (%)	OR (95% CI) for benefit receipt	p-value	AOR# (95% CI) for benefit receipt	p-value
**Overall**	220332 (100.0)	36511 (100.0)	183821 (100.0)	-		-	
**Sex$**	220332 (100.0)						
Male		16550(45.3)	92300 (50.2)	Reference		Reference	
Female		19961 (54.7)	91521 (49.8)	0.82 (0.80 to 0.84)*	p<0.001	0.78 (0.77 to 0.80)*	p<0.001
**Age (years)¥**	220329 (100.0)						
≤20		1002 (2.7)	1495 (0.8)	Reference		Reference	
21-40		18380 (50.3)	59082 (32.1)	2.15 (1.99 to 2.34)*	p<0.001	2.22 (2.04 to 2.41)*	p<0.001
41-60		14508 (39.7)	61028 (33.2)	2.82 (2.60 to 3.06)	p<0.001	2.79 (2.57 to 3.03)*	p<0.001
>60		2620 (7.2)	62214 (33.8)	15.92 (14.56 to 17.40)*	p<0.001	15.94 (14.56 to 17.46)*	p<0.001
**Ethnicity**	220332 (100.0)						
White		18403 (50.4)	106251 (57.8)	Reference		Reference	
Black/African/Caribbean/Black British		2862 (7.8)	27537 (15.0)	1.67 (1.60 to 1.74)*	p<0.001	1.98 (1.90 to 2.07)*	p<0.001
Asian/Asian British		2395 (6.6)	8387 (4.6)	0.61 (0.58 to 0.64)*	p<0.001	0.67 (0.64 to 0.71)*	p<0.001
Mixed/Multiple racial and ethnic groups		5879 (1.6)	3624 (2.0)	1.07 (0.98 to 1.17)*	p=0.138	1.73 (1.58 to 1.89)*	p<0.001
Other racial and ethnic minority groups		2850 (7.8)	9204 (5.0)	0.56 (0.53 to 0.59)*	p<0.001	0.72 (0.68 to 0.75)*	p<0.001
Not stated~		9414 (25.8)	28818 (15.7	0.53 (0.52 to 0.55)*	p<0.001	0.72 (0.70 to 0.74)*	p<0.001

For peer review only

**Death^**	220332 (100.0)						
No		34935 (95.7)	139017 (75.6)	Reference	p<0.001	Reference	p<0.001
Yes		1576 (4.3)	44804 (24.4)	7.14 (6.79 to 7.52)*	p<0.001	2.77 (2.61 to 2.93)*	p<0.001
**Deprivation (IMD quintile)≠**	211276 (95.9)						
First (most deprived)		4956 (14.2)	41296 (23.4)	Reference		Reference	
Second		12323 (35.3)	68580 (38.9)	0.67 (0.64 to 0.69)*	p<0.001	0.64 (0.61 to 0.66)*	p<0.001
Third		9013 (25.8)	37264 (21.1)	0.50 (0.48 to 0.52)*	p<0.001	0.49 (0.47 to 0.50)*	p<0.001
Fourth		5266 (15.1)	18442 (10.5)	0.42 (0.40 to 0.44)*	p<0.001	0.41 (0.39 to 0.43)*	p<0.001
Fifth (least deprived)		3404 (9.7)	10732 (6.1)	0.38 (0.36 to 0.40)*	p<0.001	0.37 (0.35 to 0.39)*	p<0.001
**Primary psychiatric diagnosis recorded∞**	220332 (100.0)						
Yes		22060 (60.4)	131702 (71.7)	1.66 (1.62 to 1.69)*	p<0.001	1.29 (1.26 to 1.33)*	p<0.001
No		14451 (39.6)	52119 (28.4)	Reference		Reference	
**Accessed IAPTΩ**	220332 (100.0)						
Yes		9707 (26.6)	41003 (22.3)	0.79 (0.77 to 0.81)*		1.01 (0.99 to 1.04)	
No		26804 (73.4)	142818 (77.7)	Reference	p<0.001	Reference	p=0.284

** P-value ≤0.01; AOR: Adjusted Odds Ratio; CI: Confidence Interval; IAPT: Improving Access to Psychological Therapies; IMD: Index of Multiple Deprivation; OR: Odds Ratio; South London and Maudsley NHS Foundation Trust. ∑ This includes patients who did not have a benefits record entry as well as those who did have an entry but did not receive any benefits; $ based on DWP data, but if missing backfilled with CRIS data; ¥ at window end date (30 June 2019), based on CRIS data; ~ includes not known, not stated or missing; ≠ IMD scores published in 2019, postcode used closest and before window end date (30 June 2019; ^ based on CRIS data, but if a death was recorded in benefits data but not recorded in CRIS data it was backfilled accordingly; ∞ latest psychiatric primary diagnosis recorded closest and before window end date (30 June 2019) based on ICD-10 F codes only (mental and behavioural disorders) but excluding non-specific diagnoses, e.g. Z*, F99*, FXX. Ω Accessed IAPT between 2008 and 30 June 2019. #AOR: adjusted for age (continuous), sex and ethnicity.*

For peer review only

Type of benefit and type of recorded psychiatric diagnosis profile

Table 4 provides an overview of the different types of benefits received among patients. Benefits most frequently reported included income replacing disability benefits (ESA, IB, DLA), unemployment benefits (JSA, UC) and IS. Many patients were also in receipt of the state pension.

Table 4: Overview of types of benefits received among linked patients (n=183,821).

**Type of benefit**µ¬	**N (%)**
Employment Support Allowance (ESA)	82436 (44.9)
Job Seekers Allowance (JSA)	75524 (41.1)
Income Support (IS)	59748 (32.5)
Disability Living Allowance (DLA)	52675 (28.7)
Incapacity Benefit (IB)	50520 (27.5)
Retirement / State Pension (RP)	49040 (26.7)
Personal Independence Pay (PIP)	47315 (25.7)
Universal Credit (UC)	46789 (25.4)
UC conditionality regime – Searching for work	38073 (81.4)
UC conditionality regime – Working, with requirements	13448 (28.7)
UC conditionality regime – No work requirements	16505 (35.3)
UC conditionality regime – Working, no requirements	13610 (29.1)
UC conditionality regime – Preparing for work	4497 (9.6)
UC conditionality regime – Planning for work	2402 (5.1)
Attendance Allowance (AA)	25017 (13.6)
Pension Credit (PC)	22749 (12.4)
Carer's Allowance (ICA)	13798 (7.5)
Severe Disablement Allowance (SDA)	3682 (2.0)
Passported Incapacity Benefit (PIB)	1622 (0.9)
Bereavement Benefit (BB)	732 (0.4)
Widows Benefit (WB)	326 (0.2)

µ benefit received between 1st of January 2005 and 30th of June 2020. ¬ PIP was only introduced in April 2013 to replace DLA. UC was only introduced in 2013. SDA was replaced by IB in April 2001. IB was replaced by ESA and since January 2011 no new IB claims have been accepted. % will not add up to 100% as patients could have received multiple benefits over time.

Most patients had a primary psychiatric diagnosis recorded in their electronic healthcare record (Table 5). About one in five patients (21.6%) were diagnosed with a mood (affective) disorder (e.g. depressive episode, mania), followed by disorders due to psychoactive substance abuse (e.g. harmful use of drugs or alcohol) (17.5%), and disorders due to physiological conditions (e.g. dementia) (17.4%). Benefit receipt across the psychiatric diagnosis spectrum was high, over 80% across most ICD-10 codes, except for behavioural syndromes associated with physiological disturbances and physical factors (56.7%) (e.g. eating disorders).

For peer review only

Table 5: Overview of recorded primary psychiatric diagnoses in linked patients (n=153,762) and whether patients who were given a diagnosis had received benefits (n=131,702).

	**Recorded primary psychiatric diagnoses∞ (ICD-10 code and description) N (%)**	**Received a benefit$^{\mu}$ N (%)**
F00-F09 (Mental and behavioural disorders, and mental disorders due to known physiological conditions)	26775 (17.4)	26069 (97.4)
F10-F19 (Mental and behavioural disorders due to psychoactive substance use)	26879 (17.5)	23731 (88.2)
F20-F29 (Schizophrenia, schizotypal, delusional disorders and other non-mood psychotic disorders)	16082 (10.5)	14944 (92.9)
F30-F39 (Mood (affective) disorders)	33235 (21.6)	27046 (81.4)
F40-F48 (Anxiety, dissociative, stress-related, somatoform and other nonpsychotic mental disorders)	25944 (16.9)	20432 (78.8)
F50-F59 (Behavioural syndromes associated with physiological disturbances and physical factors)	6773 (4.4)	3840 (56.7)
F60-F69 (Disorders of adult personality and behaviour)	6219 (4.0)	5495 (88.4)
F70-F79 (Intellectual disabilities)	2484 (1.6)	2448 (98.6)
F80-F89 (Pervasive and specific developmental disorders)	2904 (1.9)	2623 (90.3)
F90-F98 (Behavioural and emotional disorders with onset usually occurring in childhood and adolescence)	6467 (4.2)	5092 (78.7)

∞ latest psychiatric primary diagnosis recorded closest and before window end date (30 June 2019) based on ICD-10 F codes only (mental and behavioural disorders) but excluding non-specific diagnoses, e.g., Z, F99*, FXX. μ any type of benefits received between 1st of January 2005 and 30th of June 2020. % will not add up to 100% as patients could have received multiple benefits over time.*

Table 6 provides an overview of selected types of benefits received, namely those related to unemployment, sickness, disability, or income support benefits, among patients by recorded primary psychiatric diagnosis code. Most patients diagnosed with a degree of intellectual disabilities (F70-F79) were in receipt of income replacing disability benefits such as ESA and disability living allowance as well as IS and PIP. These types of benefits were also frequently received by patients diagnosed with pervasive and specific developmental disorders (e.g., disturbances in speech and language) (F80-F89)) and patients diagnosed with schizophrenia, schizotypal, delusional disorders, and other non-mood psychotic disorders (F20-F29). Unemployment benefit receipt, such as JSA, was most reported among those diagnosed with psychoactive substance abuse (63.9%). Supplementary table 1 provides an overview of the remaining benefits by recorded primary psychiatric diagnosis code and supplementary table 2 provides an overview of recorded primary psychiatric diagnosis by UC conditionality type.

Table 6: Overview of patients who had a recorded primary psychiatric diagnosis and benefit receipt related to unemployment, sickness, disability, income support benefits.

Benefit type[µ] / Recorded primary psychiatric diagnoses (ICD-10 code and description) ∞	Universal Credit (UC) N (%) n=30622	Job Seekers Allowance (JSA) N (%) n=50076	Employment Support Allowance (ESA) N (%) n=60681	Incapacity Benefit (IB) N (%) n=38336	Severe Disability Allowance (SDA) N (%) n=2957	Personal Independence Pay (PIP) N (%) n=35214	Disability Living Allowance (DLA) N (%) n=40189	Income Support (IS) N (%) n=43451
F00-F09 (Mental and behavioural disorders, and mental disorders due to known physiological conditions) n=26069	513 (2)	1352 (5.2)	2074 (8.0)	2333 (9.0)	178 (0.7)	1600 (6.1)	3734 (14.3)	1606 (6.2)
F10-F19 (Mental and behavioural disorders due to psychoactive substance use) n=23713	8574 (36.2)	15167 (64.0)	15563 (65.6)	10683 (45.1)	171 (0.7)	6165 (26.0)	4811 (20.3)	11334 (47.8)
F20-F29 (Schizophrenia, schizotypal, delusional disorders and other non-mood psychotic disorders) n=14944	2898 (19.4)	4865 (32.6)	9757 (65.3)	7176 (48.0)	975 (6.5)	6166 (41.3)	8662 (58.0)	7202 (48.2)
F30-F39 (Mood (affective) disorders) n=27046	7044 (26.0)	11351 (42.0)	12486 (46.2)	8076 (29.9)	360 (1.3)	7232 (26.7)	7840 (29.0)	9621 (35.6)
F40-F48 (Anxiety, dissociative, stress-related, somatoform and other nonpsychotic mental disorders) n=20432	5451 (26.7)	8612 (42.2)	9743 (47.7)	5543 (27.1)	199 (1.0)	6097 (29.8)	5804 (28.4)	6661 (32.6)
F50-F59 (Behavioural syndromes associated with physiological	1168 (30.4)	2124 (55.3)	1406 (36.6)	685 (17.8)	24 (0.6)	824 (21.5)	810 (21.1)	1027 (26.7)

For peer review only

disturbances and physical factors) n=3840								
F60-F69 (Disorders of adult personality and behaviour) n=5495	1874 (34.1)	2640 (48.0)	3820 (69.5)	2095 (38.1)	114 (2.1)	2615 (47.6)	2256 (41.1)	2722 (49.5)
F70-F79 (Intellectual disabilities) n=2448	238 (9.7)	246 (10.1)	1856 (75.7)	637 (26.0)	848 (34.6)	1330 (54.3)	2255 (92.1)	1451 (59.3)
F80-F89 (Pervasive and specific developmental disorders) n=2623	653 (24.9)	900 (34.3)	1598 (60.9)	448 (17.1)	66 (2.5)	1447 (55.2)	1711 (65.2)	558 (21.3)
F90-F98 (Behavioural and emotional disorders with onset usually occurring in childhood and adolescence) n-5092	2209 (43.4)	2819 (55.4)	2378 (46.7)	660 (13.0)	22 (0.4)	1738 (34.1)	2306 (45.3)	1269 (24.9)

∞ latest psychiatric primary diagnosis recorded closest and before window end date (30 June 2019) based on ICD-10 F codes only (mental and behavioural disorders) but excluding non-specific diagnoses, e.g. Z, F99*, FXX. μ any type of benefits received between 1st of January 2005 and 30th of June 2020.*

For peer review only

Discussion

We have established an unprecedented data linkage between mental healthcare and benefits records, spanning 15 years of linked data, among a substantial population of working-age adults. This enables us to look for the first time, in detail, at the complex longitudinal relationships between mental health and benefit receipt. A linkage rate of 92.3% was achieved using an ad hoc deterministic linkage approach and fuzzy matching. This high linkage rate is comparable to prior data linkages such as CRIS data with Hospital Episode Statistics and Office of National Statistics (HES-ONS) data producing a matching rate of 93.7% (25), and the CRIS data with the National Pupil Database (NPD) producing a matching rate of (82.5%) (24).

Despite the high linkage rate, there is still potential for bias, as is often the case when using an ad hoc deterministic approach where no common identifier is available between data sets. Our analysis showed that linkage bias disproportionately affected women, middle aged people, and ethnic minority groups. Women may be less likely to be linked because of changes in name and address linked to changes in relationship status, and it has been previously identified that minority groups identifiers are more likely to be entered in error and thus are particularly prone to failure of deterministic linkage processes (32, 33). We also found those with a primary psychiatric diagnosis were more likely to be linked, this may be because of having increased contact with the system and therefore increased opportunity to have personal identifiers recorded that maximise linking opportunity.

Of patients accessing SLaM services and successfully linked, 83% had engaged with the DWP, and of those, 99.8% had received a benefit of any kind. This finding is not unexpected and are in accordance with previous research showing that one of the most reported working-age disabilities and reason for claiming unemployment and sickness-related benefits is a mental health problem (1). We found those who were male, over 20 years old, had died, had a primary psychiatric diagnosis, were of a black ethnic group or mixed ethnic group and lived in a higher area of deprivation were all more likely to have received a benefit. Most received benefits among the sample included ESA, JSA and IS. Further, of those who received UC (n=46789), a high proportion were placed in the UC conditionality regime - searching for work group (n=38073, 81.4%). Next, we can explore what support and work adjustments this group are able to access in relation to finding work. We also showed that over half of the sample had received a psychiatric diagnosis, with one in five been diagnosed with a mood affective disorder. It is likely that those with a psychiatric diagnosis are more likely to fall out of work and therefore more likely to claim sickness and unemployment related benefits. A comparison of levels of benefit receipt and patterns among the UK working age population is out of scope for this paper but will be explored in detail in the future. However, we know that, for example, approximately 9.9 million working-age people were claiming a combination of benefits in 2021, including UC, PIP/DLA HB, AA, ESA, JSA , and IS (28, 29).

Previous population-based research reporting on mental health and benefit receipt has been limited in its use of self-report survey data, as well as a very basic level of detail in relation to benefit receipt. For example, the Adult Psychiatric Morbidity Survey (APMS) showed that a large proportion of people receiving ESA reported symptoms of a mental disorder, supporting our initial findings. However, the APMS did not have data on newer benefits (e.g., UC) and were unable to distinguish between the level of benefit and payment received within a particular benefit type or provide other important data such as details of the WCA process (30). Our findings are also comparable to other studies that show a large proportion of people who receive benefits report symptoms of a mental disorder (6, 7). Finally, though

For peer review only - http://bmjopen.bmj.com/site/about/guidelines.xhtml

ONS holds data reflecting labour market activity and counts of benefit claimants, mental health related data is not available (33).

There is great volume and depth of data available in this linkage. Clinical data from SLaM provides detail on both primary and secondary diagnoses, in addition to diagnosis severity as measured using the Health of the Nation Outcome Scale (HoNOS), and data on appointment history and clinical intervention provision. As SLaM is one of the largest secondary mental healthcare services in the UK, findings may be generalizable to other settings, though considerations of key differences at local level, for example type of mental healthcare services provided and the profile of patients accessing services in a highly populated, ethnically diverse urban area, should be given. In addition, SLaM provides a variety of national and specialist services, such as a specialist affective disorders service, meaning that some patients will be residing outside the SLaM catchment area. Benefits data provides extensive detail on number, type and amounts of benefits received, as well as data on interventions accessed and the WCA process. Further, the longitudinal nature of the data helps to ensure that those who engage intermittently with the welfare or mental healthcare system can still be captured where this would be more challenging in cross-sectional research or studies spanning a shorter period.

However, there are limitations of the linked data. For example, due to prior legalities, our sample includes only those who have been referred to SLaM, meaning we cannot directly compare our findings to those who have not accessed secondary mental healthcare services, but may have received benefits. In addition, as neither data set holds well populated or accurate employment related data, a proxy for returning to work is considered where someone is no longer receiving an unemployment related benefit. However, there can be varying reasons as to why someone stops receiving this type of benefit, other than because they have found work, such as no longer meeting the eligibility criteria or having a benefit suspended because of a sanction. The lack of this information may disproportionally impact vulnerable groups who are likely to have disengaged with the benefits system, such as homeless people or refugees, and still not have found work or be consistently in work. It should also be noted that interpretation of findings should consider the level of uptake and possible benefit underclaiming in the current sample (31). Notwithstanding this, the data we hold for UC, but not for other unemployment related legacy benefits provides information that indicates whether someone is in or out of work. Future projects should consider the important advantages of further linking employment related data, held by Her Majesty's Revenue and Customs in the UK, to the current linked data, as well as including a case-control population comparison group who were not referred to SLaM services.

Despite the limitations, this novel data linkage between electronic mental healthcare records and benefits records contains extensive time-variant data that allows us to look at the bidirectional and complex nature of the relationships between mental health, employment and benefit receipt, something that has not yet been possible. It provides opportunity for retrospective longitudinal cohort studies to be carried out and provide understanding of how best to design and provide the most effectively tailored interventions to target different patient groups and benefit claimants. So far, we have shown that a very high percentage of those in contact with secondary mental healthcare services have received a benefit within the 15-year window our linked data spans. We can now look in further detail at this population to answer important research questions and address areas of interest such as the impact of UC and WCA on people with mental disorders, the effectiveness of certain interventions to support people to return to work, and the general trends and trajectories of benefit receipt among

For peer review only - http://bmjopen.bmj.com/site/about/guidelines.xhtml

people accessing secondary mental healthcare services. High-quality outputs can be produced providing much needed evidence relating to both occupational and welfare policy initiatives and interventions within the DWP and NHS mental healthcare providers, all with the aim of improving outcomes for people with mental health problems.

For peer review only

For peer review only - http://bmjopen.bmj.com/site/about/guidelines.xhtml

Acknowledgements

We would like to thank Megan Pritchard at the NIHR Maudsley Biomedical Research Centre for their support with this study. We would also like to thank the members of the NIHR Maudsley Biomedical Research Nucleus Data Linkage Service User and Carer Advisory Group for their input. We are very grateful to the DWP staff, especially staff working in the Joint Health and Work Unit, who supported us in creating this linked dataset and advice provided.

Author contribution

SAMS conceptualised and designed the study with input from AP, AB, SD, NTF, MH, IM and JD. MB, RL and AJ took the lead in data curation. SAMS and AP led on the methodology, formal analysis, and project administration. MB, JD, SD, RL and AJ supported the methodology. SAMS acquired funding for the study with support from NTF, IM and MH. Supervision was provided by NTF, MH and IM. SAMS wrote the initial draft of this paper (introduction, methods, results). AP wrote the initial draft of the discussion. SAMS and AP revised the paper. All authors commented on the final draft of this paper.

Funding statement

This paper represents independent research funded by the National Institute for Health and Care Research (NIHR), as part of the corresponding author's NIHR Advanced Fellowship [ref: NIHR 300592]. This paper represents independent research part funded by the NIHR Maudsley Biomedical Research Centre at South London and Maudsley NHS Foundation Trust and King's College London [ref: N/A]. MH is a NIHR Senior Investigator. The views expressed are those of the authors and not necessarily of the NIHR, the Department of Health and Social Care or the Department for Work and Pensions.

IB is supported by the NIHR Maudsley BRC and by the NIHR Collaboration for Leadership in Applied Health Research and Care South London at King's College Hospital NHS Foundation Trust, King's College London.

Competing interests

MH is principal investigator of RADAR-CNS consortium – a public private partnership in collaboration with five pharmaceutical companies – Janssen, Biogen, UCB, MSD and Lundbeck, outside of the submitted work.

The funder had no contribution in the study design, data collection, analysis and interpretation of the data, manuscript writing and the decision to submit the paper for publications.

Patient and public involvement statement

This project was informed by discussions with the NIHR Biomedical Research Nucleus Data Linkage Service User and Carer Advisory Group.

Patient consent for publication

Not required.

Ethical approval

For peer review only - http://bmjopen.bmj.com/site/about/guidelines.xhtml

Approval has been obtained from the Health Research Authority CAG for a recommendation under s251 of the NHS Act 2006 (ref 17CAG0055), for permission to access confidential patient information without consent. The use of South London and Maudsley NHS Foundation Trust medical records data for research purposes has received approval from the NHS Research Ethics Committee (Oxford South Central ref 17/SC/0581). A data sharing agreement has been developed between the Secretary of State for Work and Pensions and the South London and Maudsley NHS Foundation Trust.

Data availability statement

Data are not publicly available. Access to deidentified data can be applied for via the NIHR Maudsley Biomedical Research Centre at the South London and Maudsley NHS Foundation Trust, upon reasonable request. Requests for data will be considered on a case-by-case basis, given the sensitive nature of the data, and access will only be granted if approval is given by the Work and Health Screening Panel and other governance requirements are fulfilled. For more information, please contact: cris.administrator@slam.nhs.uk.

For peer review only

For peer review only - http://bmjopen.bmj.com/site/about/guidelines.xhtml

References

1. Department for Work and Pensions. *Work, Health and Disability Green Paper Data Pack*. 2016.

2. Gustafsson K, Marklund S. Consequences of Sickness Presence and Sickness Absence on Health and Work Ability: A Swedish Prospective Cohort Study. *Inter J Occup Med Environ Health*. 2011;24(2):153-65.

3. de Vries H, Fishta A, Weikert B, *et al*. Determinants of Sickness Absence and Return to Work Among Employees with Common Mental Disorders: A Scoping Review. *J Occup Rehabil.* 2018;28(3):393-417.

4. Black C. *Dame Carol Black's Review of the health of Britain's working age population: working for a healthier tomorrow.* London. TSO. 2008.

5. Stevenson D, Farmer P. *Thriving at work: The Stevenson/Farmer review of mental health and employers*. 2017.

6. Sissons P, Barnes, H., Stevens, H. Routes onto Employment and Support Allowance. Department for Work and Pensions. 2011.

7. Viola S, Moncrieff J. Claims for sickness and disability benefits owing to mental disorders in the UK: trends from 1995 to 2014. *BJPsych Open*. 2016;2(1):18-24.

8. Health and Safety Executive. Working days lost in Great Britain. 2020. Available from: https://www.hse.gov.uk/statistics/dayslost.htm.

9. Adcock A, Kennedy S. *Benefit sanctions.* UK Parliament. 2015. Report No: CDP-0113.

10. Department for Work and Pensions. Universal Credit: welfare that works. 2010.

11. Barr B, Taylor-Robinson D, Stuckler D, *et al.* 'First, do no harm': are disability assessments associated with adverse trends in mental health? A longitudinal ecological study. *J Epidemiol Community Health*. 2016;70(4):339-45.

12. Barr B, Taylor-Robinson D, Stuckler D, *et al.* Fit-for-work or fit-for-unemployment? Does the reassessment of disability benefit claimants using a tougher work capability assessment help people into work? *J Epidemiol Community Health*. 2016;70(5):452-8.

13. Dwyer, P. Fi*nal findings report: Welfare Conditionality Project 2013-2018*. York: University of York; 2018.

14. House of Commons Work an Pensions Committee. *Benefit Sanctions: nineteenth special report of session 2017-19*. 2019.

15. Dwyer P, Scullion L, Jones K, *et al.* Work, welfare, and wellbeing: The impacts of welfare conditionality on people with mental health impairments in the UK. *Soc Policy Admin*. 2020;54(2):311-26.

16. Jitendra A, Thorogood E, Hadfield-Spoor M. *Left behind: is universal credit truly universal*? The Trussell Trust; 2018.

17. Department for Work and Pensions. *Simplifying the welfare system and making sure work pays.* 2015.

18. Department for Work and Pensions. *Improving Lives: The Work, Health and Disability Green Paper*. 2016.

19. Clark DM. Realizing the Mass Public Benefit of Evidence-Based Psychological Therapies: The IAPT Program. *Annu Rev Clin Psychol*. 2018;14:159-83.

20. Heffernan J, Pilkington P. Supported employment for persons with mental illness: Systematic review of the effectiveness of individual placement and support in the UK. *J Ment Health*. 2011;20(4):368-80.

21. Stewart R, Soremekun M, Perera G, *et al*. The South London and Maudsley NHS Foundation Trust Biomedical Research Centre (SLAM BRC) case register: development and descriptive data. *BMC Psychiatry*. 2009;9:51.

22. UK Government. Benefits. Available from: https://www.gov.uk/browse/benefits.

For peer review only - http://bmjopen.bmj.com/site/about/guidelines.xhtml

23. Jewell A, Pritchard M, Barrett K, *et al.* The Maudsley Biomedical Research Centre (BRC) data linkage service user and carer advisory group: creating and sustaining a successful patient and public involvement group to guide research in a complex area. *Res Involv Engagem*. 2019;5:20.
24. Downs JM, Ford T, Stewart R, *et al*. An approach to linking education, social care and electronic health records for children and young people in South London: a linkage study of child and adolescent mental health service data. *BMJ Open*. 2019;9(1).
25. Roberts E, Doidge JC, Harron KL, et al. National administrative record linkage between specialist community drug and alcohol treatment data (the National Drug Treatment Monitoring System (NDTMS)) and inpatient hospitalisation data (Hospital Episode Statistics (HES)) in England: design, method and evaluation. *BMJ Open*. 2020;10(11).
26. Minstry of Housing, Communities and Local Government. *The English Indices of Deprivation 2019*. N.d. Available from: https://assets.publishing.service.gov.uk/government/uploads/system/uploads/attachment_data/file/853811/IoD2019_FAQ_v4.pdf.
27. Department for Work and Pensions. *Dataset: People on Universal Credit.* N.d. Available from: https://stat-xplore.dwp.gov.uk/webapi/metadata/UC_Monthly/Conditionality%20Regime.html.
28. Department for Work and Pensions. *Benefit Combinations to February 2021.* 2021. Available from: https://www.gov.uk/government/statistics/dwp-benefits-statistics-august-2021/benefit-combinations-to-february-2021#working-age-combinations.
29. Department for Work and Pensions. *DWP benefits statistics: August 2021.* 2021. Available from: https://www.gov.uk/government/statistics/dwp-benefits-statistics-august-2021/dwp-benefits-statistics-august-2021.
30. McManus S, Jenkins R, Brugha T. *Mental health and wellbeing in England: Adult Psychiatric Morbidity Survey 2014.* Leeds; 2016.
31. Department for Work and Pensions. *Income-related benefits: estimates of take-up: financial year 2018 to 2019*. 2020. Available from: https://www.gov.uk/government/statistics/income-related-benefits-estimates-of-take-up-financial-year-2018-to-2019/income-related-benefits-estimates-of-take-up-financial-year-2018-to-2019.
32. Bohensk MA, Jolley D, Sundararajan V, *et al*. Data linkage: a powerful research tool with potential problems. *BMC Health Services Research*. 2010;10(1), 1-7.
33. Hagger-Johnson G, Harron K, Gonzalez-Izquierdo, *et al.* Identifying possible false matches in anonymized hospital administrative data without patient identifiers. *Health Services Research*. 2015;*50*(4), 1162-1178.
34. A guide to labour market statistics – Office for National Statistics. 2020. Available from: https://www.ons.gov.uk/employmentandlabourmarket/peopleinwork/employmentandemployeetypes/methodologies/aguidetolabourmarketstatistics.

For peer review only - http://bmjopen.bmj.com/site/about/guidelines.xhtml

Figure Legend (Figure 1)

Figure legend

IAPT: Improving Access to Psychological Therapies
SLaM: South London and Maudsley NHS Foundation Trust
DWP: Department for Work and Pensions

Figure caption

Figure 1: Overview of SLaM patient IDs that were and were not linked to benefits data from the DWP via their National Insurance Number.

For peer review only

For peer review only - http://bmjopen.bmj.com/site/about/guidelines.xhtml

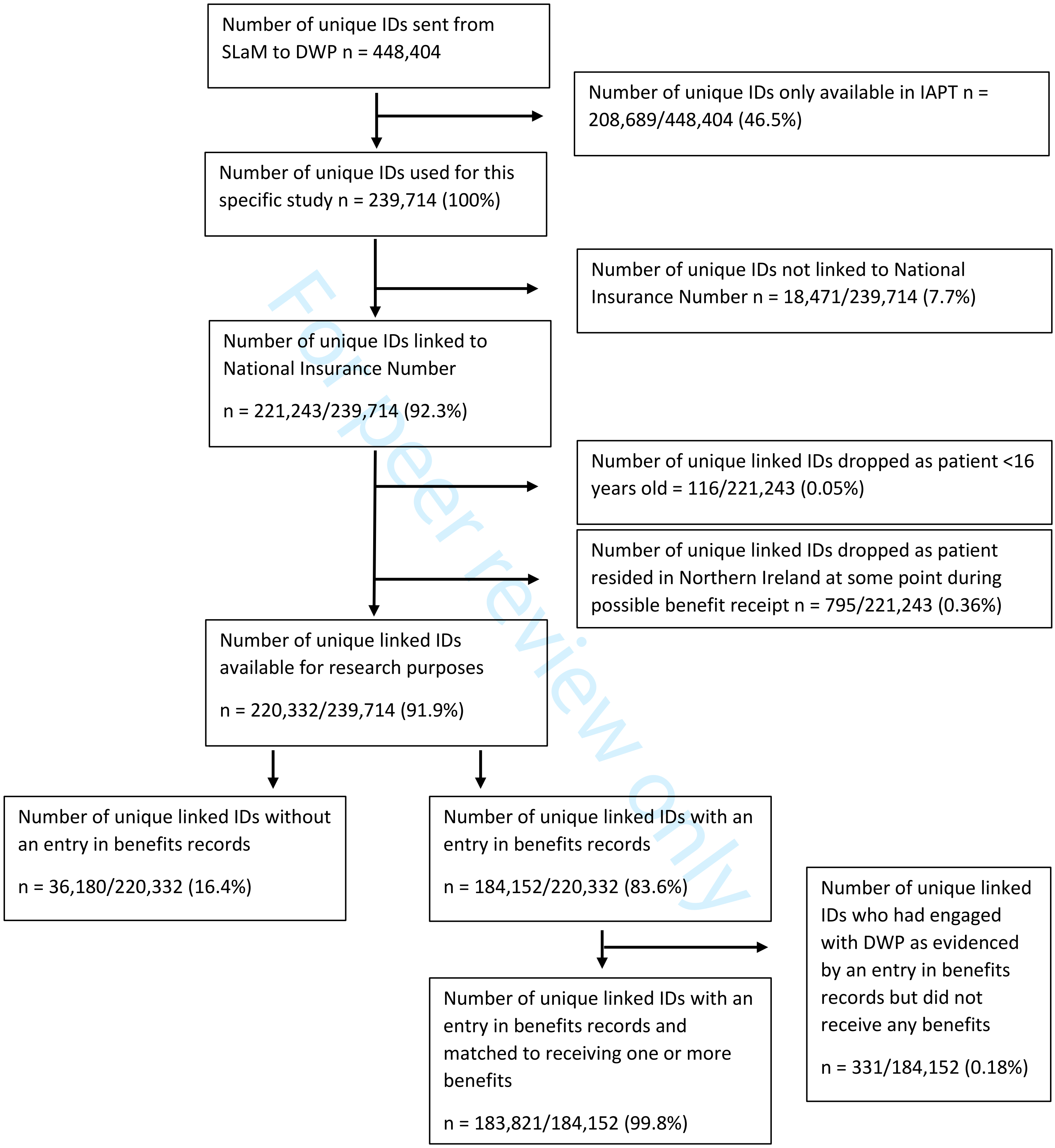

For peer review only

Supplementary Material

Data access

The DWP administrative data and CRIS clinical data are stored and hosted by the SLaM Clinical Data Linkage Service (CDLS). Researchers wishing to explore the DWP-CRIS data will first need to submit a project proposal to the CRIS Oversight Committee. The CRIS Oversight Committee will assess whether the application adherences to the agreed standards of research and dissemination specifically outlined for the CRIS database. Once approved, the application will be directed to the Work and Health Screening Panel, specifically set up to consider applications to explore the linked DWP-CRIS data. This panel is made up of a representative from the DWP and a member of the CRIS Oversight Committee. The decision to grant or deny approval for the application to access and use the linked data will be informed by the governance and ethical approvals obtained and implemented as part of the established linkage. These include: 1) NHS Health Research Authority Research Ethics Committee approval, 2) Section 251 approval under the NHS Health Research Authority Confidential Advisory Group, 3) SLaM Caldicott Guardian, 4) DWP governance panels and 5) DWP/CRIS data sharing agreement. In addition, all projects are required to have a local collaborator from King's Health Partners (e.g. SLaM, King's College London, King's College Hospital or Guy's and St Thomas' NHS Foundation Trust).

All approved projects are published with the proposal title, lay summary and lead researcher details on the public facing Maudsley BRC website (https://www.maudsleybrc.nihr.ac.uk/facilities/clinical-record-interactive-search-cris/cris-data-linkages). All research papers will be published in the CRIS publications section of the BRC website (https://www.maudsleybrc.nihr.ac.uk/facilities/clinical-record-interactive-search-cris/cris-publications/).

Once the Work and Health Screening Panel has approved the application, the applicant will work with the SLaM Clinical Data Linkage Service to develop a project data extraction specification, only including the data that is needed to answer the specific research questions as outlined in the project application. The analysis of specific extracts of the linked data will be carried out within the SLaM firewall by the applicant on site, or via a secure VPN connection. Only those who hold a contract with SLaM (substantive or honorary), or a research passport, will be able to submit a project application and work with the linked data once approved.

For peer review only

Supplementary Table 1: Overview of patients who had a recorded primary psychiatric diagnosis and had ever a benefit entry for benefits not directly related to unemployment, sickness, disability, Income Support or Universal Credit.

**Benefit type** / **Recorded primary psychiatric diagnoses (ICD-10 code and description) ∞**	**Retirement / State Pension (RP) N (%)** n=22605	**Pension Credit (PC) N (%)** n=18358	**Attendance Allowance (AA) N (%)** n=20870	**Widows Benefit (WB) N (%)** n=224	**Bereavement Benefit (BB) N (%)** n=502	**Carer's Allowance (ICA) N (%)** n=9298	**Passported Incapacity Benefit (PIB) N (%)** n=1194
F00-F09 (Mental and behavioural disorders, and mental disorders due to known physiological conditions) n=26069	22605 (86.7)	9827 (37.7)	15503 (59.5)	73 (0.3)	44 (0.2)	1146 (4.4)	32 (0.1)
F10-F19 (Mental and behavioural disorders due to psychoactive substance use) n=23713	1879 (7.9)	1118 (4.7)	413 (1.7)	19 (0.1)	68 (0.3)	2002 (8.4)	89 (0.4)
F20-F29 (Schizophrenia, schizotypal, delusional disorders and other non-mood psychotic disorders) n=14944	2732 (18.3)	2042 (13.7)	715 (4.8)	19 (0.1)	39 (0.3)	520 (3.5)	183 (1.2)
F30-F39 (Mood (affective) disorders) n=27046	6502 (24.0)	2996 (11.1)	2532 (9.4)	58 (0.2)	178 (0.7)	2426 (9.0)	122 (0.5)
F40-F48 (Anxiety, dissociative, stress-related, somatoform and other nonpsychotic mental disorders) n=20432	4128 (20.2)	1765 (8.6)	1567 (7.7)	46 (0.2)	134 (0.7)	1787 (8.8)	197 (1.0)

For peer review only

F50-F59 (Behavioural syndromes associated with physiological disturbances and physical factors) n=3840	226 (5.9)	64 (1.7)	40 (1.0)	<5 (<1.0)	18 (0.5)	276 (7.2)	50 (1.3)
F60-F69 (Disorders of adult personality and behaviour) n=5495	316 (5.8)	205 (3.7)	64 (1.2)	<5 (<1.0)	12 (0.2)	437 (8.0)	77 (1.4)
F70-F79 (Intellectual disabilities) n=2448	233 (9.5)	299 (12.2)	26 (1.1)	<5 (<1.0)	<5 (<1.0)	41 (1.7)	232 (9.5)
F80-F89 (Pervasive and specific developmental disorders) n=2623	39 (1.5)	20 (0.8)	5 (0.2)	<5 (<1.0)	<5 (<1.0)	145 (5.5)	116 (4.4)
F90-F98 (Behavioural and emotional disorders with onset usually occurring in childhood and adolescence) n-5092	59 (1.2)	22 (0.4)	5 (0.1)	<5(<1.0)	6 (0.1)	518 (10.2)	96 (1.9)

∞ latest psychiatric primary diagnosis recorded closest and before window end date (30 June 2019) based on ICD-10 F codes only (mental and behavioural disorders) but excluding non-specific diagnoses, e.g. Z, F99*, FXX. μ any type of benefits received between 1st of January 2005 and 30th of June 2020.* Cell sizes with less than <5 observations are shown as <5 (<1.0%).

For peer review only

Supplementary Table 2: Overview of patients who had a recorded primary psychiatric diagnosis and had received Universal Credit, by Universal Credit conditionality regime.

Benefit type / Recorded primary psychiatric diagnoses (ICD-10 code and description) ∞	UC Conditionality regime – searching for work (AA) N (%) n=25012	UC Conditionality regime – working, with requirements (AB) N (%) n=8409	UC Conditionality regime – no work requirements (BC) N (%) n=11404	UC Conditionality regime – working, no requirements (BD) N (%) n=8450	UC Conditionality regime – preparing for work (CE) N (%) n=2991	UC Conditionality regime – planning for work (DF) N (%) n=1488
F00-F09 (Mental and behavioural disorders, and mental disorders due to known physiological conditions) n=513	415 (80.9)	129 (25.2)	240 (46.8)	117 (22.8)	36 (7.0)	6 (1.2)
F10-F19 (Mental and behavioural disorders due to psychoactive substance use) n=8547	7605 (88.7)	1911 (22.3)	2524 (29.4)	1809 (21.1)	807 (9.4)	185 (2.2)
F20-F29 (Schizophrenia, schizotypal, delusional disorders and other non-mood psychotic disorders) n=2989	2467 (85.1)	762 (26.3)	1427 (49.2)	638 (22.0)	113 (3.9)	52 (1.8)
F30-F39 (Mood (affective) disorders) n=7044	5437 (77.2)	2212 (31.4)	2814 (40.0)	2322 (33.0)	866 (12.3)	553 (7.9)
F40-F48 (Anxiety, dissociative, stress-related, somatoform and	4197 (77.0)	1744 (32.0)	2003 (36.8)	1805 (33.1)	650 (11.9)	364 (6.7)

For peer review only

other nonpsychotic mental disorders) n=5451						
F50-F59 (Behavioural syndromes associated with physiological disturbances and physical factors) n=1168	831 (71.2)	332 (28.4)	346 (29.6)	484 (41.4)	110 (9.4)	95 (8.1)
F60-F69 (Disorders of adult personality and behaviour) n=1874	1500 (80.0)	448 (26.0)	934 (49.8)	494 (26.4)	180 (9.6)	94 (5.0)
F70-F79 (Intellectual disabilities) n=238	195 (81.9)	32 (13.5)	143 (60.1)	18 (7.6)	20 (8.4)	5 (2.1)
F80-F89 (Pervasive and specific developmental disorders) n=653	551 (84.4)	158 (24.2)	285 (43.6)	111 (17.0)	53 (8.1)	17 (2.6)
F90-F98 (Behavioural and emotional disorders with onset usually occurring in childhood and adolescence) n=2209	1814 (82.1)	641 (29.0)	688 (31.2)	652 (29.5)	156 (7.1)	117 (5.3)

∞ latest psychiatric primary diagnosis recorded closest and before window end date (30 June 2019) based on ICD 10 F codes only (mental and behavioural disorders) but excluding non-specific diagnoses, e.g. Z, F99*, FXX. µ any type of benefits received between 1st of January 2005 and 30th of June 2020.*

For peer review only

BMJ Open

Linking electronic mental healthcare and benefits records in South London: design, procedure, and descriptive outcomes.

Journal:	*BMJ Open*
Manuscript ID	bmjopen-2022-067136.R1
Article Type:	Original research
Date Submitted by the Author:	20-Nov-2022
Complete List of Authors:	Stevelink, Sharon; King's College London Institute of Psychiatry Psychology and Neuroscience, Department of Psychological Medicine; King's College London Institute of Psychiatry Psychology and Neuroscience, King's Centre for Military Health Research, Department of Psychological Medicine<br>Phillips, Ava; King's College London Institute of Psychiatry Psychology and Neuroscience, Department of Psychological Medicine<br>Broadbent, Matthew; NIHR Maudsley Biomedical Research Centre, South London and Maudsley Mental Health NHS Trust<br>Boyd, Andy; University of Bristol Medical School, Population Health Sciences<br>Dorrington, Sarah; King's College London Institute of Psychiatry Psychology and Neuroscience; NIHR Maudsley Biomedical Research Centre, South London and Maudsley Mental Health NHS Trust<br>Jewell, Amelia; NIHR Maudsley Biomedical Research Centre, South London and Maudsley Mental Health NHS Trust<br>Leal, Ray; King's College London Institute of Psychiatry Psychology and Neuroscience, King's Centre for Military Health Research, Department of Psychological Medicine; King's College London Institute of Psychiatry Psychology and Neuroscience, Department of Psychological Medicine<br>Bakolis, Ioannis; King's College London Institute of Psychiatry Psychology and Neuroscience, Health Services and Population Research Department; King's College London Institute of Psychiatry Psychology and Neuroscience, Department of Biostatistics and Health Informatics<br>MADAN, IRA; Guy's and St Thomas' Hospitals NHS Trust, Department of Occupational Health; King's College London<br>Hotopf, Matthew; King's College London Institute of Psychiatry Psychology and Neuroscience, Department of Psychological Medicine; NIHR Maudsley Biomedical Research Centre, South London and Maudsley Mental Health NHS Trust<br>Fear, Nicola; King's College London Institute of Psychiatry Psychology and Neuroscience, King's Centre for Military Health Research, Department of Psychological Medicine; King's College London Institute of Psychiatry Psychology and Neuroscience, Academic Department of Military Mental Health, Department of Psychological Medicine<br>Downs, Johnny; King's College London Institute of Psychiatry Psychology and Neuroscience, Department of Psychological Medicine; NIHR Maudsley Biomedical Research Centre, South London and Maudsley Mental Health NHS Trust
Primary Subject	Epidemiology

Heading:	
Secondary Subject Heading:	Public health, Epidemiology, Occupational and environmental medicine
Keywords:	Epidemiology < TROPICAL MEDICINE, MENTAL HEALTH, Public health < INFECTIOUS DISEASES

SCHOLARONE™ Manuscripts

I, the Submitting Author has the right to grant and does grant on behalf of all authors of the Work (as defined in the below author licence), an exclusive licence and/or a non-exclusive licence for contributions from authors who are: i) UK Crown employees; ii) where BMJ has agreed a CC-BY licence shall apply, and/or iii) in accordance with the terms applicable for US Federal Government officers or employees acting as part of their official duties; on a worldwide, perpetual, irrevocable, royalty-free basis to BMJ Publishing Group Ltd ("BMJ") its licensees and where the relevant Journal is co-owned by BMJ to the co-owners of the Journal, to publish the Work in this journal and any other BMJ products and to exploit all rights, as set out in our licence.

The Submitting Author accepts and understands that any supply made under these terms is made by BMJ to the Submitting Author unless you are acting as an employee on behalf of your employer or a postgraduate student of an affiliated institution which is paying any applicable article publishing charge ("APC") for Open Access articles. Where the Submitting Author wishes to make the Work available on an Open Access basis (and intends to pay the relevant APC), the terms of reuse of such Open Access shall be governed by a Creative Commons licence – details of these licences and which Creative Commons licence will apply to this Work are set out in our licence referred to above.

Other than as permitted in any relevant BMJ Author's Self Archiving Policies, I confirm this Work has not been accepted for publication elsewhere, is not being considered for publication elsewhere and does not duplicate material already published. I confirm all authors consent to publication of this Work and authorise the granting of this licence.

For peer review only

Linking electronic mental healthcare and benefits records in South London: design, procedure, and descriptive outcomes.

Sharon A.M. Stevelink# [1,2], Ava Phillips# [1], Matthew Broadbent [3], Andy Boyd [4], Sarah Dorrington [1,3], Amelia Jewell [3], Ray Leal [1,2], Ioannis Bakolis [5,6], Ira Madan [8], Matthew Hotopf [1,3], Nicola T. Fear* [2,9], Johnny Downs* [1,3].

# joint first authors

* Joint last authors

1. Department of Psychological Medicine, Institute of Psychiatry, Psychology and Neuroscience, King's College London, London, UK.
2. King's Centre for Military Health Research, Department of Psychological Medicine, Institute of Psychiatry, Psychology and Neuroscience, King's College London, London, UK.
3. NIHR Maudsley Biomedical Research Centre, South London and Maudsley Mental Health NHS Trust, London, UK.
4. Population Health Sciences, Bristol Medical School, University of Bristol, Bristol, UK.
5. Centre for Implementation Science, Health Services and Population Research Department, Institute of Psychiatry, Psychology and Neuroscience, London, UK.
6. Department of Biostatistics and Health Informatics, Institute of Psychiatry, Psychology and Neuroscience, London, United Kingdom.
7. Department of Occupational Health, Guy's and St Thomas' Hospitals NHS Trust, London, UK.
8. King's College London, London, UK.
9. Academic Department of Military Mental Health, Department of Psychological Medicine, Institute of Psychiatry, Psychology and Neuroscience, King's College London, London, UK.

Corresponding author: Dr Sharon Stevelink, Institute of Psychiatry, Psychology and Neuroscience, King's College London, 16 De Crespigny Park, London, UK, SE5 9AF.

Email: Sharon.stevelink@kcl.ac.uk

Word count: 5060

Key words: benefits, data linkage, Department for Work and Pensions, epidemiology, electronic healthcare records, mental health, public health, South London and Maudsley NHS Foundation Trust, welfare state.

For peer review only - http://bmjopen.bmj.com/site/about/guidelines.xhtml

Abstract

Objectives: To describe the process and outcomes of a data linkage between electronic secondary mental healthcare records from the South London and Maudsley (SLaM) NHS Foundation Trust with benefits records from the Department for Work and Pensions (DWP). We also describe the mental health and benefit profile of patients who were successfully linked.

Design: A deterministic linkage of routine records from health and welfare government service providers within a secure environment.

Setting and participants: Adults aged ≥18 years who were referred to or accessed treatment at SLaM services between January 2007 and June 2019, including those who were treated as part of Improving Access to Psychological Therapies (IAPT) services between January 2008 and June 2019 (n=448,404). Benefits data from the DWP from January 2005 until June 2020.

Outcome measures: The linkage rate and associated socio-demographic, diagnostic and treatment factors. Recorded primary psychiatric diagnosis based on International Classification of Diseases (ICD)-10 codes and type of benefit receipt.

Results: A linkage rate of 92.3% was achieved. Women, younger patients, and those from ethnic minority groups were less likely to be successfully linked. Patients who had subsequently died, had a recorded primary psychiatric diagnosis, had also engaged with IAPT, and had a higher number of historical postcodes available were more likely to be linked. Eighty-three percent of patients received benefits at some point between 2005 and 2020. Benefit receipt across the psychiatric diagnosis spectrum was high, over 80% across most ICD-10 codes.

Conclusions: This data linkage is the first of its kind in the UK demonstrating the use of routinely collected mental health and benefits data. Benefit receipt was high among patients accessing SLaM services and varied by psychiatric diagnosis. Future areas of research are discussed, including exploring the effectiveness of interventions for helping people into work, and the impact of benefit reforms.

For peer review only - http://bmjopen.bmj.com/site/about/guidelines.xhtml

Summary

- This is a novel data linkage between electronic mental healthcare records and benefits records in the UK.
- A strength of this data linkage is the high linkage rate of 92.3%.
- The sample does not include a comparison group (e.g., people who did not access secondary mental healthcare services).
- There is no reliable employment variable within the data stating whether someone is currently in or out of work (except for Universal Credit).
- There is a potential for linkage bias as a result of the method used (ad hoc deterministic fuzzy matching) and having no unique identifier between data sets.

For peer review only

For peer review only - http://bmjopen.bmj.com/site/about/guidelines.xhtml

Introduction

In the UK, approximately 1.8 million people face long-term sickness absence of four weeks or longer, costing our society £100 billion annually (1). Long-term sickness absence is associated with social exclusion, poor health outcomes and high mortality (2-4). Each year, over 300,000 people are leaving work due to long-term mental health problems (5). Mental disorders are one of the most common causes of sickness absence and subsequent long-term occupational disability (6, 7). In 2019/2020, 17.9 million working days were lost due to mental ill health (8). For many who access mental health services, their difficulties impact on their ability to work. Understanding people's finances, welfare, benefits, and occupational needs are integral to the care and quality of life for people with mental disorders, however these are often overlooked.

Over the last 15 years, major changes have taken place in the UK benefits system including the extension of benefit sanctions (9); the introduction of 'Universal Credit' (UC), a means-tested benefit replacing six benefits plus tax credits for those of working-age (10); the replacement of personal capability assessments with work capability assessments (WCA) where one's capability for all work-related activity is reviewed; and an increased reliance on conditionality meaning that people need to fulfil certain work-related activity requirements to maintain their full benefit entitlements. These were announced as part of the *Welfare Reform Act 2007* and *2012*, and *Welfare Reform and Work Act 2016*. These changes have been met with concern about their potential impact on people's well-being, and particularly on those with mental disorders (11-16). Hence, research into the welfare and benefit needs of the population with mental disorders is required, to inform policy on welfare provision when this group is at their most vulnerable; also to support return to work as an integral part of recovery for people who are able to return to employment (17, 18). The latter is especially relevant given the introduction of, for example, Improving Access to Psychological Therapies services (19) and Individual Placement and Support Services (20) in the UK.

There are no pre-existing datasets in the UK that can currently address this. Alone, NHS healthcare records are an unreliable source of information on benefit receipts or employment status; these are not routinely collected or recorded. Data held by the Department for Work and Pensions (DWP) which records national welfare and public service interactions, for example on unemployment-related benefits, lacks high-quality information about health status. The limited data that is available in these benefits records are solely based on diagnostic information provided in benefit applications for specific benefits, and these are often incomplete.

The advent of electronic healthcare records and systems, and the increasing sophistication with which data can be linked and analysed, has presented the opportunity to change the academic research landscape. We report here on a unique linkage of welfare and benefits data with routinely collected mental health data of over 400,000 adults referred to psychiatric services, enabling us to address gaps in evidence regarding the interrelationships between benefit receipt, employment status, mental disorders, treatment, well-being and recovery. To our knowledge, this is the first time in the UK that routine health records have been linked with benefits data. However, research into welfare and mental health using data registries have been led by those in Nordic countries where a unique personal identifier is available to all those with a permanent residence record, paving the way for opportunities in linkages between health and welfare registers (21-23).

For peer review only - http://bmjopen.bmj.com/site/about/guidelines.xhtml

Here, we describe the process and outcomes of linking electronic mental healthcare records from patients who accessed secondary mental healthcare services at the South London and Maudsley (SLaM) NHS Foundation Trust with benefits records from the DWP. First, we will describe the ethical and governance considerations encountered before we could proceed with the linkage. Second, we describe the approach, data linkage rate and factors associated with successful linkage. Finally, we provide an overview of the mental health and benefit profile of patients who were successfully linked.

Methods

Data sources

South London and Maudsley NHS Foundation Trust Biomedical Research Centre Case Register

The SLaM NHS Foundation Trust is one of Europe's largest providers of secondary mental healthcare services, providing care predominantly for the South London boroughs of Lambeth, Lewisham, Southwark, and Croydon, covering a catchment area of over 1.2 million residents. SLaM provides specialist (secondary) mental healthcare services as well as Improving Access to Psychological Therapies (IAPT) services. The SLaM Biomedical Research Centre (BRC) Case Register includes electronic mental healthcare records of patients accessing SLaM. In 2008, the Clinical Records Interactive Search (CRIS) system was developed (24) to curate deidentified data from SLaM's electronic mental healthcare records for research use. Information concerning patients' mental healthcare journey is available in pseudo-anonymised format either in free clinical text notes or structured fields as part of a patient's electronic mental healthcare record. CRIS clinical data may include, for example, individual level data on socio-demographic characteristics (e.g. month and year of birth, sex, ethnicity, neighbourhood deprivation), time variant data on International Classification of Diseases (ICD)-10 psychiatric diagnosis, diagnostic assessments, mental health treatment (e.g. local or specialist services, community vs. inpatient), service use (e.g. patterns of engagement), medication prescriptions and psychotherapeutic interventions. For the current paper, only data from structured fields were used. CRIS data covered the 1st of January 2007 till the 30th of June 2019.

Department for Work and Pensions benefits data

The DWP is responsible for the implementation of policy regarding most welfare and state benefits in Great Britain. Benefits data includes individual level demographic data (e.g. date of birth, date of death, and sex), time variant data related to the on and off flows of benefits (e.g. Incapacity Benefit, Carers Allowance, Income Support, Jobseeker's Allowance, Attendance Allowance, Retirement/State Pension, Disability Living Allowance, Severe Disablement Benefit, Widow's Benefit, Pension Credit, Passported Incapacity Benefit, Bereavement Benefit, Employment and Support Allowance, Universal Credit, Personal Independence Pay and relevant benefit specific details) (25). Start and end dates of benefit spells are provided as well as the amount of money received. In addition, some information is provided about WCA and work programme participation. Benefits data covered 1st of January 2005 till 30th of June 2020.

Sample

For peer review only - http://bmjopen.bmj.com/site/about/guidelines.xhtml

The sample consists of all adults (aged 18 years and older) who 1) have been referred for treatment with SLaM secondary mental healthcare services between 1st January 2007 (the implementation of electronic mental healthcare records across SLaM secondary mental healthcare services was only finalised by that time) and 30th June 2019, or 2) had an event with SLaM secondary mental healthcare services during this time period and were aged 18 or over at the time of their latest recorded event in the window, or 3) had a treatment episode at IAPT between 1st January 2008 to 30th June 2019. Patients ranged in symptom severity from common mental disorders to serious mental illness (e.g. schizophrenia, bipolar affective disorder), substance use disorders and organic disorders (e.g. neurological syndromes associated with severe intellectual impairment). For the current paper, we only focused on the linkage of patients who accessed specialist (secondary) mental healthcare services within SLaM (and possibly also IAPT) but not those who only accessed IAPT within SLaM. This decision was made as we were especially interested in the former group of patients who were more likely to have severe mental health symptomatology.

Patient and public involvement and engagement

The proposed linkage of electronic mental healthcare records of SLaM and benefits records from the DWP was presented to the Maudsley Biomedical Research Centre Data Linkage Service User and Carer Advisory Group in December 2016 (26). The members of the Advisory Group experienced mental ill health themselves or as a carer for someone with a mental health diagnosis and were accessing or had accessed mental healthcare services. All were given training concerning data linkages, the underlying clinical research information system, data security, governance, and the research environment at SLaM.

The members of the Advisory Group were supportive of the proposed linkage when first discussed in December 2016. The linkage was presented again in September 2019 with a discussion around the specific research questions and opportunities for continued patient and public involvement in the project. They will be consulted on a regular basis now the data linkage has been finalised with a focus on discussing preliminary results and gathering input regarding dissemination and impact strategies.

Ethical and governance approvals

We submitted the proposed linkage to the South Central – Oxford C Research Ethics Committee for ethical approval. A favourable opinion was received in 2017 (ref 17/SC/0581). In addition, we successfully applied in 2017 for Section 251 approval under the NHS Health Research Authority Confidential Advisory Group (ref 17CAG0055). We believed that it was not practical or appropriate for the proposed linkage to be successfully achieved through a consent-based methodology.

Once ethical approvals were in place, we developed a data sharing agreement. This agreement outlines the data sharing agreements between SLaM and the Department for Work and Pensions in relation to the data linkage. The agreement sets out lawful basis of the data linkage as well as the principles and procedures for data sharing and the use of the linked data. Details on how to access the linked data can be found in the Supplementary Material (Supplement 1).

Data linkage process

The linkage of CRIS clinical records with benefits data took place in late 2020. An ad hoc deterministic matching approach was used, namely fuzzy matching, based on personal identifiers held on the DWP's

For peer review only - http://bmjopen.bmj.com/site/about/guidelines.xhtml

Customer Information System (CIS) which hosts a 'spine' record of everyone who has ever been issued a National Insurance Number (NINO). The NINO is a unique individual ID allocated for employment, tax, and welfare purposes.

1. The SLaM Clinical Data Linkage Service, 'a trusted third party', shared the personal identifiers of the eligible sample (patient name, date of birth, sex, postcode and postcode history) and the BRCID pseudonym used within the CRIS database with DWP (the data were transferred using the secure 'Egress' system).
2. The DWP linked the SLaM personal identifiers to DWP held personal identifiers in a secure area using a fuzzy-matching process (uniqueness cut-off threshold of 90% or above) to create a table linking the BRCID pseudonym to a NINO (where possible). Approved benefits data were extracted from DWP systems using the NINO.
3. The NINO was replaced with the BRCID pseudonym before the linked de-identified DWP benefits data were sent back to the SLaM Clinical Data Linkage Service via Egress. At no point were SLaM clinical data shared. DWP destroyed the SLaM personal identifiers once the matching work was complete.
4. The benefits data with the attached BRCIDs are stored within the SLaM secure research system in a separate database to the CRIS clinical data with access to restricted users only.
5. The benefits data and CRIS clinical data are only joined on a project specific basis, after the necessary approvals have been given. BRCIDs are stripped before a project specific anonymised data set is provided to the researcher.

Materials

The following socio-demographic and clinical, diagnostic and treatment variables were derived from the linked data for further exploration. These were selected based on data availability, previous research indicating that these factors were found to be associated with data linkage success (27, 28), and discussions within the wider research team.

Socio-demographic variables

All socio-demographic variables were derived from the clinical data, except for patient sex (male/female) as this was more complete in the benefits data. However, if sex was missing in the benefits data, and available in the clinical data, this was backfilled accordingly. Age was calculated using month and year of birth until the SLaM window end date (30th June 2019). Subsequently, age was grouped in the following categories: ≤20, 21-40, 41-60 and >60. Ethnicity was categorised as follows: White /Black, African, Caribbean, Black British/ Asian, Asian British/Mixed, Multiple racial and ethnic groups/ Other racial and ethnic minority groups and 'not stated'. We also had information on whether people had died (month and year) that resulted in a binary death (yes/no) variable. The Index of Multiple Deprivation (IMD) was informed by 2019 data, and we used the postcode closest to and before the SLaM window end date to inform IMD quintiles, with the first quintile indicating most deprived and fifth quintile least deprived. IMD is a summary measure of relative deprivation informed by 7 domains, namely income, employment, education, crime, housing, health and living environment at lower levels of geography (29). We created a variable indicating whether patients lived in the local catchment area based on Lower-layer Super Output Areas (LSOA11), a small geographical area covering a similar population size, again using the postcode closest to and before the SLaM window

For peer review only - http://bmjopen.bmj.com/site/about/guidelines.xhtml

end date (29). In addition, we generated a categorical variable indicating the number of historical postcodes sent to DWP to facilitate the linkage for each patient (up to five maximum).

Diagnostic and treatment variables

We created a binary primary psychiatric diagnosis variable (yes/no) that referred to whether a psychiatric primary diagnosis was recorded in a patient's record closest and before the SLaM window end date (30th June 2019). This only included the ICD 10 'F codes' referring to mental and behavioural disorders, thereby excluding non-specific diagnoses (e.g. Z*, F99*, FXX). Subsequently, we derived a variable outlining the type of diagnosis code patients were given, if any (ranging from F00-F09 (Mental and behavioural disorders, and mental disorders due to known physiological conditions) to F90-F98 (Behavioural and emotional disorders with onset usually occurring in childhood and adolescence). We also explored whether patients had accessed IAPT (yes/no), in addition to SLaM services between 2008 and 30th June 2019. IAPT was only introduced in 2008 so this was the earliest possible start date. Two binary variables were created (before and after 2013) to indicate patients' first and last contact with SLaM. This cut off was chosen as Personal Independence Payment was introduced in 2013. Age at first presentation to SLaM (≤20, 21-40, 41-60, >60) was calculated using month and year of birth and the patients' earliest accepted referral date to SLaM closest to and before the SLaM window end date.

Benefits variables

Participants who were successfully linked to a NINO and had received one of the following benefits between 1st of January 2005 till 30th of June 2020 were identified as benefit recipients: Employment and Support Allowance (ESA), Jobseeker's Allowance (JSA), Income Support (IS), Disability Living Allowance (DLA), Incapacity Benefit (IB), Retirement/State Pension (RP), Personal Independence Payment (PIP), Universal Credit (UC), Pension Credit (PC), Carer's Allowance (ICA), Severe Disablement Allowance (SDA), Passported Incapacity Benefit (PIB) or Widow's Benefits (WB) (25). We also had information on what UC conditionality regime patients were allocated to namely 1) searching for work, 2) working, with requirements, 3) no work requirements 4) working, no requirements, 5) preparing for work, or 6) planning for work (30).

Statistical analysis

Analysis of linkage bias

All statistical analyses were performed using the statistical package STATA (version 15). All variables were checked for completeness and outliers. Variable completeness and accuracy were improved by backfilling data (using the clinical or benefits records were possible). If outliers were identified, for example date of birth (as based on the age inclusion criteria), this was recoded as missing (n=14). The same was done for negative values (e.g., age at first contact n=192) and improbable dates (e.g., having accessed SLaM before it was established n=2210).

The overall linkage rate was determined by calculating the proportion of unique BRCIDs successfully linked to a NINO. We did not expect all patients to have engaged with the DWP to apply for benefits or subsequently successfully received benefits. For example, some participants engaged with the DWP, and a note was made on their benefits record, but they did not meet the criteria to receive, for example, Employment and Support Allowance. Therefore, of those successfully linked to a NINO, we

For peer review only - http://bmjopen.bmj.com/site/about/guidelines.xhtml

For peer review only

also calculated the proportion who had engaged with the DWP, as well as the proportion who had engaged and successfully applied for benefits according to the benefits records.

We then conducted univariable logistic regression analysis to explore socio-demographic, diagnostic and treatment related factors, associated with linkage to benefits records. We also conducted multivariable analyses thereby adjusting for factors identified *a priori* (namely age, sex and ethnicity) (24, 25). Subsequently, we generated a probability estimate of matching as a function of the risk variables with the use of the logistic regression model.

Sample profile

Multivariable logistic regression models were also employed to explore factors associated with benefit receipt, adjusting for age, sex and ethnicity. In addition, descriptive statistics were used to describe the benefit and the mental health profile of successfully linked patients. The latter was based on the most recently recorded ICD-10 primary psychiatric diagnostic code. We also tabulated the mental health profile of our sample by type of benefit receipt. Odds Ratios (OR), Adjusted Odds Ratios (AOR), 95% Confidence Intervals (CI) and p-values are reported.

Results

Overview of data linkage process and analysis of linkage bias

Unique IDs of 448,404 patients who accessed SLaM services (specialist (secondary) mental healthcare services and/or IAPT) were sent to the DWP (Figure 1). For this study, we only report on patients who accessed secondary mental healthcare services at SLaM (n=239,714). Of these, 221,243 (92.3%) were successfully linked to a NINO held by the DWP. Individuals identified as being under the age of 16 according to the personal details held by the DWP and those who resided in Northern Ireland[1] at some point during benefit receipt were excluded from the data sent back to the SLaM Clinical Data Linkage Service, resulting in 220,332 (91.9%) unique linked IDs available for research purposes.

[1] The Health Research Authority approval that was received for the data linkage only applies to England and Wales. In addition, the devolved legislature of Northern Ireland is responsible for administering benefits to patients who resided in Northern Ireland at the time of their benefit receipt. Therefore, the DWP do not have authority to share this data.

For peer review only - http://bmjopen.bmj.com/site/about/guidelines.xhtml

Results from adjusted logistic regression analyses indicated that the following groups of patients were less likely to be linked (an OR greater than 1 denotes greater chance of successful linkage compared with the reference): female patients vs. male patients, ethnic minority groups vs. patients from a white ethnic background, and patients with only one postcode available vs. two or more postcodes. Compared to younger patients (<21 years), middle-aged patients (21-60 years) were less likely to be successfully linked, whereas older patients (>60 years) were more likely to be linked compared to all other age groups (Table 1). We also found that those who had died, had a recorded psychiatric primary diagnosis, had engaged with IAPT and accessed SLaM services more recently were more likely to be successfully linked (Table 2).

Socio-demographic, diagnostic and treatment related factors associated with benefit receipt

Of the patients who were successfully linked, 184,152 (83.6%) had engaged with the DWP, meaning they had a benefits record but not necessarily successfully claimed benefits. Among the successfully linked patients who had engaged, 183,821 (99.8%) had received benefits at some point between the 1st January 2005 and 30th June 2020 (Table 3). Adjusted results indicated that benefit receipt was higher among men, those over the age of 20 years compared with younger patients, those who had subsequently died, had a recorded primary psychiatric diagnosis and patients living in an area of higher deprivation. Patients from a black ethnic group and those from a mixed ethnic group were more likely to report benefit receipt compared to patients from other ethnic backgrounds.

For peer review only - http://bmjopen.bmj.com/site/about/guidelines.xhtml

Table 1: Comparison of socio-demographic characteristics of linked and unlinked patients with benefits data (n=239,714).

	Total N (%)	Linked N (%)	Non-linked N (%)	OR (95% CI) for successful linkage	p-value	AOR# (95% CI) for successful linkage	p-value
**Overall**	239714 (100.0)	221243 (100.0)	18471 (100.0)	-			
**Sex**[$]	239690 (100.0)						
Male		109321 (49.4)	8215 (44.5)	Reference		Reference	
Female		111921 (50.6)	10233 (55.5)	0.82 (0.80 to 0.85)*	p<0.001	0.81 (0.79 to 0.84)*	p<0.001
**Age (years)**[¥]	239699 (100.0)						
≤20		2502 (1.1)	142 (0.8)	Reference		Reference	
21-40		77943 (35.2)	9033 (48.9)	0.49 (0.41 to 0.58)*	p<0.001	0.49 (0.42 to 0.59)*	p<0.001
41-60		75860 (34.3)	6839 (37.0)	0.63 (0.53 to 0.75)*	p<0.001	0.62 (0.52 to 0.73)*	p<0.001
>60		64935 (29.4)	2445 (13.3)	1.51 (1.27 to 1.79)*	p<0.001	1.40 (1.17 to 1.66)*	p<0.001
**Ethnicity**	239714 (100.0)						
White		125244 (56.6)	7405 (40.1)	Reference		Reference	
Black/African/Caribbean/Black British		30464 (13.8)	3495 (18.9)	0.52 (0.49 to 0.54)*	p<0.001	0.56 (0.53 to 0.58)*	p<0.001
Asian/Asian British		10812 (4.9)	1708 (9.3)	0.37 (0.35 to 0.40)*	p<0.001	0.40 (0.38 to 0.42)*	p<0.001
Mixed/Multiple racial and ethnic groups		4225 (1.9)	346 (1.9)	0.72 (0.65 to 0.81)*	p<0.001	0.93 (0.83 to 1.04)*	p=0.177
Other racial and ethnic minority groups		12099 (5.5)	1889 (10.2)	0.38 (0.36 to 0.40)*	p<0.001	0.44 (0.42 to 0.46)*	p<0.001
Not stated[˜]		38399 (17.4)	3628 (19.6)	0.63 (0.60 to 0.65)*	p<0.001	0.74 (0.71 to 0.78)*	p<0.001
**Death**[^]	239714 (100.0)						
No		174820 (79.0)	17063 (92.4)	Reference		Reference	

For peer review only - http://bmjopen.bmj.com/site/about/guidelines.xhtml

Yes		46423 (21.0)	1408 (7.6)	3.22 (3.04 to 3.40)*	p<0.001	1.91 (1.79 to 2.03)*	p<0.001
**Deprivation (IMD quintile)≠**	227755 (95.0)						
First (most deprived)		46403 (21.9)	3390 (21.6)	Reference		Reference	
Second		81207 (38.3)	6536 (41.7)	0.91 (0.87 to 0.95)*	p<0.001	0.90 (0.86 to 0.94)*	p<0.001
Third		46443 (21.9)	3546 (22.6)	0.96 (0.91 to 1.00)	p=0.076	0.92 (0.87 to 0.96)*	p=0.001
Fourth		23774 (11.2)	1430 (9.1)	1.21 (1.14 to 1.29)*	p<0.001	1.09 (1.02 to 1.19)*	P=0.012
Fifth (least deprived)		14165 (6.7)	779 (5.0)	1.33 (1.23 to 1.44)*	p<0.001	1.14 (1.05 to 1.24)*	P=0.001
**Resident within local catchment area π**	227997 (95.0)						
Yes		146860 (69.2)	11177 (71.2)	1.06 (1.02 to 1.11)*	p<0.001	1.03 (0.99 to 1.08)*	p<0.001
No		65435 (30.8)	4525 (28.8)	Reference		Reference	
**Number of home/residential postcodes available¬**	236412 (98.6)						
1		118603 (54.2)	10374 (59.6)	Reference		Reference	
2		47538 (21.7)	3474 (20.0)	1.20 (1.15 to 1.25)*	p<0.001	1.23 (1.19 to 1.29)*	p<0.001
3		22252 (10.2)	1497 (8.6)	1.30 (1.23 to 1.38)*	p<0.001	1.39 (1.32 to 1.48)*	p<0.001
4		11733 (5.4)	813 (4.7)	1.26 (1.17 to 1.36)*	p<0.001	1.41 (1.31 to 1.52)*	p<0.001
5		18885 (8.6)	1243 (7.1)	1.33 (1.25 to 1.41)*	p<0.001	1.57 (1.47 to 1.67)*	p<0.001

** P-value ≤0.01; AOR: Adjusted Odds Ratio; CI: Confidence Interval; IMD: Index of Multiple Deprivation; OR: Odds Ratio; $ based on DWP data, but if missing backfilled with SLaM data ¥ at window end date (30 June 2019), based on CRIS data; ~ includes not known, not stated or missing; ≠ IMD scores published in 2019, postcode used closest and before window end date (30 June 2019); π based on Lower-layer Super Output Areas (LSOA11) informed by postcode details closest to and before window end date (30 June 2019); ^ based on CRIS data, but if a death was recorded in benefits data but not recorded in CRIS data it was backfilled accordingly; ¬ based on five closest postcodes to the earliest accepted referral into SLaM or event within the time window; #AOR: adjusted for age (continuous), sex and ethnicity.*

For peer review only - http://bmjopen.bmj.com/site/about/guidelines.xhtml

For peer review only

Table 2: Comparison of diagnostic and treatment characteristics of linked and unlinked patients with benefits data (n=239,714).

	Total N (%)	Linked N (%)	Non-linked N (%)	OR (95% CI) for successful linkage	p-value	AOR# (95% CI) for successful linkage	p-value
**Overall**	239714 (100.0)	221243 (100.0)	18471 (100.0)	-			
**Primary psychiatric diagnosis recorded∞**	239714 (100.0)						
Yes		154354 (69.8)	10997 (59.5)	1.57 (1.52 to 1.62)*	p<0.001	1.43 (1.38 to 1.48)*	p<0.001
No		66889 (30.2)	7474 (40.5)	Reference		Reference	p<0.001
**Accessed IAPTΩ**	239714 (100.0)						
Yes		50899 (23.0)	3381 (18.3)	1.33 (1.28 to 1.39)*	p<0.001	1.69 (1.63 to 1.76)*	p<0.001
No		170344 (77.0)	15090 (81.7)	Reference		Reference	
**First contact with SLaM**	233186 (97.3)						
Before 2013		121339 (56.4)	10,989 (61.4)	Reference		Reference	
After 2012		93936 (43.6)	6922 (38.7)	1.23 (1.19 to 1.27)*	p<0.001	1.45 (1.40 to 1.50)*	p<0.001
**Last contact with SLaM**	235396 (98.4)						
Before 2013		73945 (34.0)	8486 (47.2)	Reference		Reference	
After 2012		143474 (66.0)	9491 (52.8)	1.73 (1.68 to 1.79)*	p<0.001	2.10 (2.04 to 2.19)*	p<0.001
**Age (years) at first presentation to SLaM**	235204 (98.1)						
≤20		23926 (11.0)	2106 (11.7)	Reference		Reference	
21-40		92178 (42.4)	10834 (60.3)	0.75 (0.71 to 0.79)*	p<0.001	0.67 (0.63 to 0.71)*	p<0.001
41-60		55388 (25.5)	3593 (20.0)	1.36 (1.28 to 1.43)*	p<0.001	0.98 (0.90 to 1.07)	p=0.637
>60		45754 (21.1)	1427 (8.0)	2.82 (2.63 to 3.02)*	p<0.001	1.53 (1.33 to 1.76)*	p<0.001

For peer review only - http://bmjopen.bmj.com/site/about/guidelines.xhtml

** P-value ≤0.01; AOR: Adjusted Odds Ratio; CI: Confidence Interval; IAPT: Improving Access to Psychological Therapies; OR: Odds Ratio; ∞ latest psychiatric primary diagnosis recorded closest and before window end date (30 June 2019) based on ICD-10 F codes only (mental and behavioural disorders) but excluding non-specific diagnoses, e.g. Z*, F99*, FXX. Ω Accessed IAPT between 2008 and 30 June 2019. #AOR: adjusted for age (continuous), sex and ethnicity.*

For peer review only - http://bmjopen.bmj.com/site/about/guidelines.xhtml

Table 3: Overview of characteristics of those who did or did not ever receive any benefits among linked patients (n=220,332).

	Total N (%)	Never received benefits$^{\Sigma}$ N (%)	Ever received benefits N (%)	OR (95% CI) for benefit receipt	p-value	AOR# (95% CI) for benefit receipt	p-value
**Overall**	220332 (100.0)	36511 (100.0)	183821 (100.0)	-		-	
**Sex$^{\$}$**	220332 (100.0)						
Male		16550(45.3)	92300 (50.2)	Reference		Reference	
Female		19961 (54.7)	91521 (49.8)	0.82 (0.80 to 0.84)*	p<0.001	0.78 (0.77 to 0.80)*	p<0.001
**Age (years)$^{¥}$**	220329 (100.0)						
≤20		1002 (2.7)	1495 (0.8)	Reference		Reference	
21-40		18380 (50.3)	59082 (32.1)	2.15 (1.99 to 2.34)*	p<0.001	2.22 (2.04 to 2.41)*	p<0.001
41-60		14508 (39.7)	61028 (33.2)	2.82 (2.60 to 3.06)	p<0.001	2.79 (2.57 to 3.03)*	p<0.001
>60		2620 (7.2)	62214 (33.8)	15.92 (14.56 to 17.40)*	p<0.001	15.94 (14.56 to 17.46)*	p<0.001
**Ethnicity**	220332 (100.0)						
White		18403 (50.4)	106251 (57.8)	Reference		Reference	
Black/African/Caribbean/Black British		2862 (7.8)	27537 (15.0)	1.67 (1.60 to 1.74)*	p<0.001	1.98 (1.90 to 2.07)*	p<0.001
Asian/Asian British		2395 (6.6)	8387 (4.6)	0.61 (0.58 to 0.64)*	p<0.001	0.67 (0.64 to 0.71)*	p<0.001
Mixed/Multiple racial and ethnic groups		5879 (1.6)	3624 (2.0)	1.07 (0.98 to 1.17)*	p=0.138	1.73 (1.58 to 1.89)*	p<0.001
Other racial and ethnic minority groups		2850 (7.8)	9204 (5.0)	0.56 (0.53 to 0.59)*	p<0.001	0.72 (0.68 to 0.75)*	p<0.001
Not stated~		9414 (25.8)	28818 (15.7	0.53 (0.52 to 0.55)*	p<0.001	0.72 (0.70 to 0.74)*	p<0.001

**Death^**	220332 (100.0)						
No		34935 (95.7)	139017 (75.6)	Reference	p<0.001	Reference	p<0.001
Yes		1576 (4.3)	44804 (24.4)	7.14 (6.79 to 7.52)*	p<0.001	2.77 (2.61 to 2.93)*	p<0.001
**Deprivation (IMD quintile)≠**	211276 (95.9)						
First (most deprived)		4956 (14.2)	41296 (23.4)	Reference		Reference	
Second		12323 (35.3)	68580 (38.9)	0.67 (0.64 to 0.69)*	p<0.001	0.64 (0.61 to 0.66)*	p<0.001
Third		9013 (25.8)	37264 (21.1)	0.50 (0.48 to 0.52)*	p<0.001	0.49 (0.47 to 0.50)*	p<0.001
Fourth		5266 (15.1)	18442 (10.5)	0.42 (0.40 to 0.44)*	p<0.001	0.41 (0.39 to 0.43)*	p<0.001
Fifth (least deprived)		3404 (9.7)	10732 (6.1)	0.38 (0.36 to 0.40)*	p<0.001	0.37 (0.35 to 0.39)*	p<0.001
**Primary psychiatric diagnosis recorded∞**	220332 (100.0)						
Yes		22060 (60.4)	131702 (71.7)	1.66 (1.62 to 1.69)*	p<0.001	1.29 (1.26 to 1.33)*	p<0.001
No		14451 (39.6)	52119 (28.4)	Reference		Reference	
**Accessed IAPTΩ**	220332 (100.0)						
Yes		9707 (26.6)	41003 (22.3)	0.79 (0.77 to 0.81)*		1.01 (0.99 to 1.04)	
No		26804 (73.4)	142818 (77.7)	Reference	p<0.001	Reference	p=0.284

** P-value ≤0.01; AOR: Adjusted Odds Ratio; CI: Confidence Interval; IAPT: Improving Access to Psychological Therapies; IMD: Index of Multiple Deprivation; OR: Odds Ratio; South London and Maudsley NHS Foundation Trust. ∑ This includes patients who did not have a benefits record entry as well as those who did have an entry but did not receive any benefits; $ based on DWP data, but if missing backfilled with CRIS data; ¥ at window end date (30 June 2019), based on CRIS data; ~ includes not known, not stated or missing; ≠ IMD scores published in 2019, postcode used closest and before window end date (30 June 2019; ^ based on CRIS data, but if a death was recorded in benefits data but not recorded in CRIS data it was backfilled accordingly; ∞ latest psychiatric primary diagnosis recorded closest and before window end date (30 June 2019) based on ICD-10 F codes only (mental and behavioural disorders) but excluding non-specific diagnoses, e.g. Z*, F99*, FXX. Ω Accessed IAPT between 2008 and 30 June 2019. #AOR: adjusted for age (continuous), sex and ethnicity.*

For peer review only

Recorded psychiatric diagnosis profile and benefit receipt

Most patients had a primary psychiatric diagnosis recorded in their electronic healthcare record (Table 4). About one in five patients (21.6%) were diagnosed with a mood (affective) disorder (e.g. depressive episode, mania), followed by disorders due to psychoactive substance abuse (e.g. harmful use of drugs or alcohol) (17.5%), and disorders due to physiological conditions (e.g. dementia) (17.4%). Benefit receipt across the psychiatric diagnosis spectrum was high, over 80% across most ICD-10 codes, except for behavioural syndromes associated with physiological disturbances and physical factors (56.7%) (e.g. eating disorders). Of those receiving benefits, 85.1% received 2 or more different benefits.

For peer review only

For peer review only - http://bmjopen.bmj.com/site/about/guidelines.xhtml

Table 4: Overview of recorded primary psychiatric diagnoses in linked patients (n=153,762) and whether patients who were given a diagnosis had received benefits (n=131,702).

	**Recorded primary psychiatric diagnoses∞ (ICD-10 code and description) N (%)**	**Received a benefit$^{\mu}$ N (%)**
F00-F09 (Mental and behavioural disorders, and mental disorders due to known physiological conditions)	26775 (17.4)	26069 (97.4)
F10-F19 (Mental and behavioural disorders due to psychoactive substance use)	26879 (17.5)	23731 (88.2)
F20-F29 (Schizophrenia, schizotypal, delusional disorders and other non-mood psychotic disorders)	16082 (10.5)	14944 (92.9)
F30-F39 (Mood (affective) disorders)	33235 (21.6)	27046 (81.4)
F40-F48 (Anxiety, dissociative, stress-related, somatoform and other nonpsychotic mental disorders)	25944 (16.9)	20432 (78.8)
F50-F59 (Behavioural syndromes associated with physiological disturbances and physical factors)	6773 (4.4)	3840 (56.7)
F60-F69 (Disorders of adult personality and behaviour)	6219 (4.0)	5495 (88.4)
F70-F79 (Intellectual disabilities)	2484 (1.6)	2448 (98.6)
F80-F89 (Pervasive and specific developmental disorders)	2904 (1.9)	2623 (90.3)
F90-F98 (Behavioural and emotional disorders with onset usually occurring in childhood and adolescence)	6467 (4.2)	5092 (78.7)

∞ latest psychiatric primary diagnosis recorded closest and before window end date (30 June 2019) based on ICD-10 F codes only (mental and behavioural disorders) but excluding non-specific diagnoses, e.g., Z, F99*, FXX. μ any type of benefits received between 1st of January 2005 and 30th of June 2020. % will not add up to 100% as patients could have received multiple benefits over time.*

Table 5 provides an overview of selected types of benefits received, namely those related to unemployment, sickness, disability, or income support benefits, among patients by recorded primary psychiatric diagnosis code. Most patients diagnosed with a degree of intellectual disabilities (F70-F79) were in receipt of disability benefits such as ESA and DLA or income support benefits such as IS and PIP. These types of benefits were also frequently received by patients diagnosed with pervasive and specific developmental disorders (e.g., disturbances in speech and language) (F80-F89)) and patients diagnosed with schizophrenia, schizotypal, delusional disorders, and other non-mood psychotic disorders (F20-F29). Unemployment benefit receipt, such as JSA, was most reported among those diagnosed with psychoactive substance abuse (63.9%). Supplementary table 1 provides an overview of the types of benefits received among the linked patients irrespective of recorded psychiatric diagnosis code, supplementary table 2 provides an overview of the remaining benefits (e.g. RP, PC, AA, WB, BB, ICA, PIB) by recorded primary psychiatric diagnosis code and supplementary table 3 provides an overview of recorded primary psychiatric diagnosis by UC conditionality type.

For peer review only - http://bmjopen.bmj.com/site/about/guidelines.xhtml

Table 5: Overview of patients who had a recorded primary psychiatric diagnosis and benefit receipt related to unemployment, sickness, disability, income support benefits.

**Benefit type**[µ] / **Recorded primary psychiatric diagnoses (ICD-10 code and description)** ∞	**Universal Credit (UC) N (%)** n=30622	**Jobeeker's Allowance (JSA) N (%)** n=50076	**Employment and Support Allowance (ESA) N (%)** n=60681	**Incapacity Benefit (IB) N (%)** n=38336	**Severe Disability Allowance (SDA) N (%)** n=2957	**Personal Independence Payment (PIP) N (%)** n=35214	**Disability Living Allowance (DLA) N (%)** n=40189	**Income Support (IS) N (%)** n=43451
F00-F09 (Mental and behavioural disorders, and mental disorders due to known physiological conditions) n=26069	513 (2.0)	1352 (5.2)	2074 (8.0)	2333 (9.0)	178 (0.7)	1600 (6.1)	3734 (14.3)	1606 (6.2)
F10-F19 (Mental and behavioural disorders due to psychoactive substance use) n=23713	8574 (36.2)	15167 (64.0)	15563 (65.6)	10683 (45.1)	171 (0.7)	6165 (26.0)	4811 (20.3)	11334 (47.8)
F20-F29 (Schizophrenia, schizotypal, delusional disorders and other non-mood psychotic disorders) n=14944	2898 (19.4)	4865 (32.6)	9757 (65.3)	7176 (48.0)	975 (6.5)	6166 (41.3)	8662 (58.0)	7202 (48.2)
F30-F39 (Mood (affective) disorders) n=27046	7044 (26.0)	11351 (42.0)	12486 (46.2)	8076 (29.9)	360 (1.3)	7232 (26.7)	7840 (29.0)	9621 (35.6)
F40-F48 (Anxiety, dissociative, stress-related, somatoform and other nonpsychotic mental disorders) n=20432	5451 (26.7)	8612 (42.2)	9743 (47.7)	5543 (27.1)	199 (1.0)	6097 (29.8)	5804 (28.4)	6661 (32.6)
F50-F59 (Behavioural syndromes associated with physiological	1168 (30.4)	2124 (55.3)	1406 (36.6)	685 (17.8)	24 (0.6)	824 (21.5)	810 (21.1)	1027 (26.7)

For peer review only

disturbances and physical factors) n=3840								
F60-F69 (Disorders of adult personality and behaviour) n=5495	1874 (34.1)	2640 (48.0)	3820 (69.5)	2095 (38.1)	114 (2.1)	2615 (47.6)	2256 (41.1)	2722 (49.5)
F70-F79 (Intellectual disabilities) n=2448	238 (9.7)	246 (10.1)	1856 (75.7)	637 (26.0)	848 (34.6)	1330 (54.3)	2255 (92.1)	1451 (59.3)
F80-F89 (Pervasive and specific developmental disorders) n=2623	653 (24.9)	900 (34.3)	1598 (60.9)	448 (17.1)	66 (2.5)	1447 (55.2)	1711 (65.2)	558 (21.3)
F90-F98 (Behavioural and emotional disorders with onset usually occurring in childhood and adolescence) n-5092	2209 (43.4)	2819 (55.4)	2378 (46.7)	660 (13.0)	22 (0.4)	1738 (34.1)	2306 (45.3)	1269 (24.9)

∞ latest psychiatric primary diagnosis recorded closest and before window end date (30 June 2019) based on ICD-10 F codes only (mental and behavioural disorders) but excluding non-specific diagnoses, e.g. Z, F99*, FXX. µ any type of benefits received between 1st of January 2005 and 30th of June 2020.*

For peer review only

Discussion

We have established an unprecedented data linkage between routinely collected mental healthcare and benefits records, spanning 15 years of linked data, among working-age adults. This enables us to look for the first time, in detail, at the complex longitudinal relationships between mental health and benefit receipt. A linkage rate of 92.3% was achieved using an ad hoc deterministic linkage approach and fuzzy matching. This high linkage rate is comparable to prior data linkages such as CRIS data with Hospital Episode Statistics and Office of National Statistics (HES-ONS) data producing a matching rate of 93.7% (31), and the CRIS data with the National Pupil Database (NPD) producing a matching rate of (82.5%) (27).

Despite the high linkage rate, there is still potential for bias, as is often the case when using an ad hoc deterministic approach where no common identifier is available between data sets. Our analysis showed that linkage bias disproportionately affected women, middle aged people, and ethnic minority groups. Women may be less likely to be linked because of changes in name and address linked to changes in relationship status, and it has been previously identified that minority groups identifiers are more likely to be entered in error and thus are particularly prone to failure of deterministic linkage processes (32, 33). We also found those with a primary psychiatric diagnosis were more likely to be linked, this may be because of having increased contact with the system and therefore increased opportunity to have personal identifiers recorded that maximise linking opportunity.

Of patients accessing SLaM services and successfully linked, 83% had engaged with the DWP, and of those, 99.8% had received a benefit of any kind. This finding is not unexpected and are in accordance with previous research showing that one of the most reported working-age disabilities and reason for claiming unemployment and sickness-related benefits is a mental health problem (1). We found those who were male, over 20 years old, had subsequently died, had a primary psychiatric diagnosis, were of a black ethnic group or mixed ethnic group and lived in a higher area of deprivation were all more likely to have received a benefit. Most received benefits among the sample included ESA, JSA and IS. Further, of those who received UC (n=46789), a high proportion were placed in the UC conditionality regime - searching for work group (n=38073, 81.4%). Next, we can explore what support and work adjustments this group are able to access in relation to finding work. We also showed that over half of the sample had received a psychiatric diagnosis, with one in five been diagnosed with a mood affective disorder. It is likely that those with a psychiatric diagnosis are more likely to fall out of work and therefore more likely to claim sickness and unemployment related benefits. A comparison of levels of benefit receipt and patterns among the UK working age population is out of scope for this paper but will be explored in detail in the future. However, we know that, for example, approximately 9.9 million working-age people were claiming a combination of benefits in 2021, including UC, PIP/DLA HB, AA, ESA, JSA , and IS (34, 35).

Previous population-based research reporting on mental health and benefit receipt in the UK has been limited in its use of self-report survey data, as well as a basic level of detail in relation to benefit receipt. For example, the Adult Psychiatric Morbidity Survey (APMS) (2014) showed that a large proportion of people receiving ESA reported symptoms of a mental disorder, supporting our initial findings. Nevertheless, the APMS did not have data on newer benefits (e.g., UC) and were unable to distinguish between the level of benefit and payment received within a particular benefit type or provide other important data such as details of the WCA process (36). Our findings are also comparable to other studies that show a large proportion of people who receive benefits report

For peer review only - http://bmjopen.bmj.com/site/about/guidelines.xhtml

symptoms of a mental disorder (6, 7). Finally, ONS holds data reflecting labour market activity and collects information via the Labour Force Survey (LFS) relating to (un)employment, counts of benefit claimants, and selected self-reported physical and mental health conditions. However detailed, longitudinal health data is not available (37).

Though we are yet to explore sickness and disability related benefits among our sample in detail, much research into disability pension (DP) awards has already been conducted in Norway using large population-based cohorts containing mental and physical health data linked to national databases of disability benefits using national identification numbers. For example, one study investigated the impact of anxiety and depression on DPs awarded for mental health and physical health diagnoses. They showed long-term occupational impact of anxiety and depression symptoms and their subsequent independent contribution towards DPs awarded (23). Another study linking mental health cohort data and the National Insurance Administration database containing DP award data showed that anxiety and depression at baseline were strongly associated with receiving a DP award at follow-up (22). A Finnish study found that there was evidence of regional variation in mental disorder DP and mental health service factors, a critical finding when considering service provision (21).

There is great volume and depth of data available in the newly established linkage. Clinical data from SLaM provides detail on both primary and secondary diagnoses, in addition to diagnosis severity as measured using the Health of the Nation Outcome Scale (HoNOS), and data on appointment history and clinical intervention provision. As SLaM is one of the largest secondary mental healthcare services in the UK, findings may be generalizable to other settings, though considerations of key differences at local level, for example type of mental healthcare services provided and the profile of patients accessing services in a highly populated, ethnically diverse urban area, should be given. In addition, SLaM provides a variety of national and specialist services, such as a specialist affective disorders service, meaning that some patients will be residing outside the SLaM catchment area. Benefits data provides extensive detail on number, type and amounts of benefits received, as well as data on interventions accessed and the WCA process. Further, the longitudinal nature of the data helps to ensure that those who engage intermittently with the welfare or mental healthcare system can still be captured where this would be more challenging in cross-sectional research or studies spanning a shorter period.

However, there are limitations of the linked data. For example, due to prior legalities, our sample includes only those who have been referred to SLaM, meaning we cannot directly compare our findings to those who have not accessed secondary mental healthcare services, but may have received benefits. In addition, as neither data set holds well populated or accurate employment related data, a proxy for returning to work is considered where someone is no longer receiving an unemployment related benefit. However, there can be varying reasons as to why someone stops receiving this type of benefit, other than because they have found work, such as no longer meeting the eligibility criteria or having a benefit suspended because of a sanction. The lack of this information may disproportionally impact vulnerable groups who are likely to have disengaged with the benefits system, such as homeless people or refugees, and still not have found work or be consistently in work. It should also be noted that interpretation of findings should consider the level of uptake and possible benefit underclaiming in the current sample (38). Notwithstanding this, the data we hold for UC, but not for other unemployment related legacy benefits provides information that indicates whether someone is in or out of work. Future projects should consider the important advantages of further

For peer review only - http://bmjopen.bmj.com/site/about/guidelines.xhtml

linking employment related data, held by Her Majesty's Revenue and Customs in the UK, to the current linked data, as well as including a case-control population comparison group who were not referred to SLaM services.

Despite the limitations, this novel data linkage between routinely collected electronic mental healthcare records and benefits records contains extensive time-variant data that allows us to explore the bidirectional and complex relationships between mental health, employment and benefit receipt, something that has not previously been seen in the UK. It provides opportunity for retrospective longitudinal cohort studies to be carried out and provide understanding of how best to design and provide the most effectively tailored interventions to target different patient groups and benefit claimants. So far, we have shown that a very high percentage of those in contact with secondary mental healthcare services have received a benefit at some point within the 15-year window our linked data spans. We can now look in further detail at this population to answer important research questions and address areas of interest such as the impact of UC and WCA on people with mental disorders, the effectiveness of certain interventions to support people to return to work, and the general trends and trajectories of benefit receipt among people accessing secondary mental healthcare services. High-quality outputs can be produced providing much needed evidence relating to both occupational and welfare policy initiatives and interventions within the joint DWP/Department of Health and Social Care Work and Health Unit, and NHS mental healthcare providers, all with the aim of improving outcomes for people with mental health problems.

For peer review only - http://bmjopen.bmj.com/site/about/guidelines.xhtml

Acknowledgements

We would like to thank Megan Pritchard at the NIHR Maudsley Biomedical Research Centre for their support with this study. We would also like to thank the members of the NIHR Maudsley Biomedical Research Nucleus Data Linkage Service User and Carer Advisory Group for their input. We are very grateful to the DWP/Department of Health and Social Care joint Work and Health Unit staff, especially staff working in the Joint Health and Work Unit, who supported us in creating this linked dataset and advice provided.

Author contribution

SAMS conceptualised and designed the study with input from AP, AB, SD, IB, NTF, MH, IM and JD. MB, RL and AJ took the lead in data curation. SAMS and AP led on the methodology, formal analysis, and project administration. MB, JD, SD, RL, IB and AJ supported the methodology. SAMS acquired funding for the study with support from NTF, IM and MH. Supervision was provided by NTF, MH and IM. SAMS wrote the initial draft of this paper (introduction, methods, results). AP wrote the initial draft of the discussion. SAMS and AP revised the paper. All authors commented on the final draft of this paper.

Funding statement

This paper represents independent research funded by the National Institute for Health and Care Research (NIHR), as part of the corresponding author's NIHR Advanced Fellowship [ref: NIHR 300592]. This paper represents independent research part funded by the NIHR Maudsley Biomedical Research Centre at South London and Maudsley NHS Foundation Trust and King's College London [ref: N/A]. MH is a NIHR Senior Investigator. The views expressed are those of the authors and not necessarily of the NIHR, the Department of Health and Social Care or the Department for Work and Pensions.

IB is supported by the NIHR Maudsley BRC and by the NIHR Collaboration for Leadership in Applied Health Research and Care South London at King's College Hospital NHS Foundation Trust, King's College London.

Competing interests

MH is principal investigator of RADAR-CNS consortium – a public private partnership in collaboration with five pharmaceutical companies – Janssen, Biogen, UCB, MSD and Lundbeck, outside of the submitted work.

The funder had no contribution in the study design, data collection, analysis and interpretation of the data, manuscript writing and the decision to submit the paper for publications.

Patient and public involvement statement

This project was informed by discussions with the NIHR Biomedical Research Nucleus Data Linkage Service User and Carer Advisory Group.

Patient consent for publication

Not required.

Ethical approval

For peer review only - http://bmjopen.bmj.com/site/about/guidelines.xhtml

Approval has been obtained from the Health Research Authority CAG for a recommendation under s251 of the NHS Act 2006 (ref 17CAG0055), for permission to access confidential patient information without consent. The use of South London and Maudsley NHS Foundation Trust medical records data for research purposes has received approval from the NHS Research Ethics Committee (Oxford South Central ref 17/SC/0581). A data sharing agreement has been developed between the Department for Work and Pensions and the South London and Maudsley NHS Foundation Trust.

Data availability statement

Data are not publicly available. Access to deidentified data can be applied for via the NIHR Maudsley Biomedical Research Centre at the South London and Maudsley NHS Foundation Trust, upon reasonable request. Requests for data will be considered on a case-by-case basis, given the sensitive nature of the data, and access will only be granted if approval is given by the Work and Health Screening Panel and other governance requirements are fulfilled. For more information, please contact: cris.administrator@slam.nhs.uk.

For peer review only

For peer review only - http://bmjopen.bmj.com/site/about/guidelines.xhtml

References

1. Department for Work and Pensions. *Work, Health and Disability Green Paper Data Pack*. 2016.

2. Gustafsson K, Marklund S. Consequences of Sickness Presence and Sickness Absence on Health and Work Ability: A Swedish Prospective Cohort Study. *Inter J Occup Med Environ Health*. 2011;24(2):153-65.

3. de Vries H, Fishta A, Weikert B, *et al*. Determinants of Sickness Absence and Return to Work Among Employees with Common Mental Disorders: A Scoping Review. *J Occup Rehabil.* 2018;28(3):393-417.

4. Black C. *Dame Carol Black's Review of the health of Britain's working age population: working for a healthier tomorrow.* London. TSO. 2008.

5. Stevenson D, Farmer P. *Thriving at work: The Stevenson/Farmer review of mental health and employers*. 2017.

6. Sissons P, Barnes, H., Stevens, H. Routes onto Employment and Support Allowance. Department for Work and Pensions. 2011.

7. Viola S, Moncrieff J. Claims for sickness and disability benefits owing to mental disorders in the UK: trends from 1995 to 2014. *BJPsych Open*. 2016;2(1):18-24.

8. Health and Safety Executive. Working days lost in Great Britain. 2020. Available from: https://www.hse.gov.uk/statistics/dayslost.htm.

9. Adcock A, Kennedy S. *Benefit sanctions.* UK Parliament. 2015. Report No: CDP-0113.

10. Department for Work and Pensions. Universal Credit: welfare that works. 2010.

11. Barr B, Taylor-Robinson D, Stuckler D, *et al.* 'First, do no harm': are disability assessments associated with adverse trends in mental health? A longitudinal ecological study. *J Epidemiol Community Health*. 2016;70(4):339-45.

12. Barr B, Taylor-Robinson D, Stuckler D, *et al.* Fit-for-work or fit-for-unemployment? Does the reassessment of disability benefit claimants using a tougher work capability assessment help people into work? *J Epidemiol Community Health*. 2016;70(5):452-8.

13. Dwyer, P. *Final findings report: Welfare Conditionality Project 2013-2018*. York: University of York; 2018.

14. House of Commons Work an Pensions Committee. *Benefit Sanctions: nineteenth special report of session 2017-19*. 2019.

15. Dwyer P, Scullion L, Jones K, *et al.* Work, welfare, and wellbeing: The impacts of welfare conditionality on people with mental health impairments in the UK. *Soc Policy Admin*. 2020;54(2):311-26.

16. Jitendra A, Thorogood E, Hadfield-Spoor M. *Left behind: is universal credit truly universal*? The Trussell Trust; 2018.

17. Department for Work and Pensions. *Simplifying the welfare system and making sure work pays.* 2015.

18. Department for Work and Pensions. *Improving Lives: The Work, Health and Disability Green Paper*. 2016.

19. Clark DM. Realizing the Mass Public Benefit of Evidence-Based Psychological Therapies: The IAPT Program. *Annu Rev Clin Psychol*. 2018;14:159-83.

20. Heffernan J, Pilkington P. Supported employment for persons with mental illness: Systematic review of the effectiveness of individual placement and support in the UK. *J Ment Health*. 2011;20(4):368-80.

21. Karolaakso T, Autio R, Näppilä T, *et al*. Contextual and mental health service factors in mental-disorder based disability pensioning in Finland - a regional comparison. *BMC Health Serv Res.* 2021; 21(1), 1-13.

22. Mykeltun A, Overland S, Dahl A, *et al.* A population-based cohort study of the effects of common mental disorders on disability pension awards. *American J Psych.* 2006; 163(8), 1412-1418.

For peer review only - http://bmjopen.bmj.com/site/about/guidelines.xhtml

23. Knudsen AK, Øverland S, Aakvaag HF, *et al.* Common mental disorders and disability pension award: seven-year follow-up study of the HUSK study. *J Psychosom Res.* 2010; 69(1), 59-67.
24. Stewart R, Soremekun M, Perera G, *et al*. The South London and Maudsley NHS Foundation Trust Biomedical Research Centre (SLAM BRC) case register: development and descriptive data. *BMC Psychiatry*. 2009;9:51.
25 UK Government. Benefits. Available from: https://www.gov.uk/browse/benefits.
26. Jewell A, Pritchard M, Barrett K, *et al.* The Maudsley Biomedical Research Centre (BRC) data linkage service user and carer advisory group: creating and sustaining a successful patient and public involvement group to guide research in a complex area. *Res Involv Engagem*. 2019;5:20.
27. Downs JM, Ford T, Stewart R, *et al*. An approach to linking education, social care and electronic health records for children and young people in South London: a linkage study of child and adolescent mental health service data. *BMJ Open*. 2019;9(1).
28. Roberts E, Doidge JC, Harron KL, et al. National administrative record linkage between specialist community drug and alcohol treatment data (the National Drug Treatment Monitoring System (NDTMS)) and inpatient hospitalisation data (Hospital Episode Statistics (HES)) in England: design, method and evaluation. *BMJ Open*. 2020;10(11).
29. Minstry of Housing, Communities and Local Government. *The English Indices of Deprivation 2019*. N.d. Available from: https://assets.publishing.service.gov.uk/government/uploads/system/uploads/attachment_data/file/853811/IoD2019_FAQ_v4.pdf.
30. Department for Work and Pensions. *Dataset: People on Universal Credit.* N.d. Available from: https://stat-xplore.dwp.gov.uk/webapi/metadata/UC_Monthly/Conditionality%20Regime.html.
31. Jewell A, Broadbent M, Hayes RD, *et al.* Impact of matching error on linked mortality outcome in a data linkage of secondary mental health data with Hospital Episode Statistics (HES) and mortality records in South East London: a cross-sectional study. *BMJ Open.* 2020;10(7), e035884.
32. Department for Work and Pensions. *Benefit Combinations to February 2021.* 2021. Available from: https://www.gov.uk/government/statistics/dwp-benefits-statistics-august-2021/benefit-combinations-to-february-2021#working-age-combinations.
33. Department for Work and Pensions. *DWP benefits statistics: August 2021.* 2021. Available from: https://www.gov.uk/government/statistics/dwp-benefits-statistics-august-2021/dwp-benefits-statistics-august-2021.
34. McManus S, Jenkins R, Brugha T. *Mental health and wellbeing in England: Adult Psychiatric Morbidity Survey 2014.* Leeds; 2016.
35. Department for Work and Pensions. *Income-related benefits: estimates of take-up: financial year 2018 to 2019*. 2020. Available from: https://www.gov.uk/government/statistics/income-related-benefits-estimates-of-take-up-financial-year-2018-to-2019/income-related-benefits-estimates-of-take-up-financial-year-2018-to-2019.
36. Bohensk MA, Jolley D, Sundararajan V, *et al*. Data linkage: a powerful research tool with potential problems. *BMC Health Services Research*. 2010;10(1), 1-7.
37. Hagger-Johnson G, Harron K, Gonzalez-Izquierdo, *et al.* Identifying possible false matches in anonymized hospital administrative data without patient identifiers. *Health Services Research*. 2015;*50*(4), 1162-1178.
38. A guide to labour market statistics – Office for National Statistics. 2020. Available from: https://www.ons.gov.uk/employmentandlabourmarket/peopleinwork/employmentandemployeetypes/methodologies/aguidetolabourmarketstatistics.

For peer review only - http://bmjopen.bmj.com/site/about/guidelines.xhtml

Figure Legend (Figure 1)

Figure legend

IAPT: Improving Access to Psychological Therapies
SLaM: South London and Maudsley NHS Foundation Trust
DWP: Department for Work and Pensions

Figure caption

Figure 1: Overview of SLaM patient IDs that were and were not linked to benefits data from the DWP via their National Insurance Number.

For peer review only

For peer review only - http://bmjopen.bmj.com/site/about/guidelines.xhtml

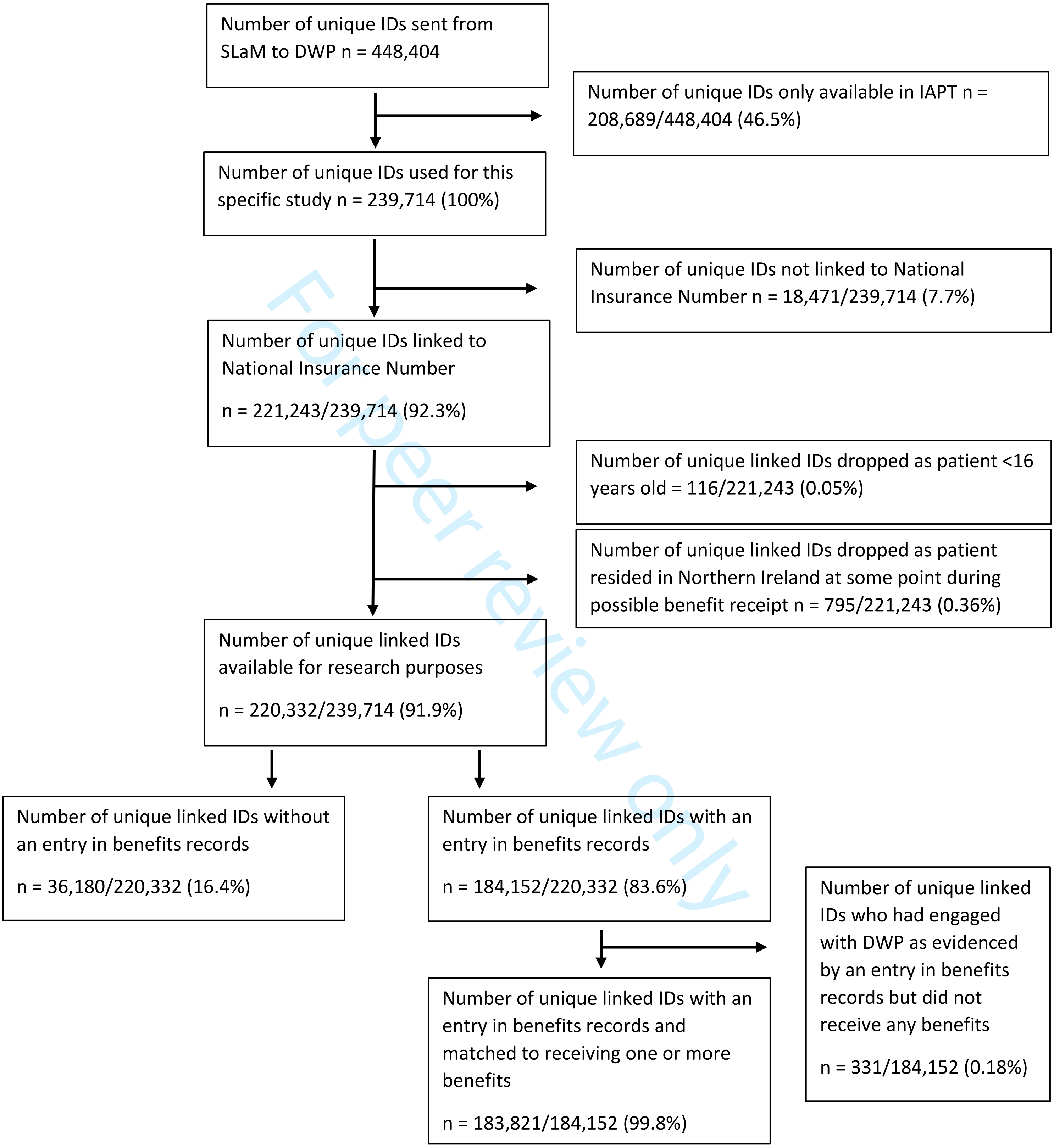

For peer review only

Supplementary Material

Data access

The DWP administrative data and CRIS clinical data are stored and hosted by the SLaM Clinical Data Linkage Service (CDLS). Researchers wishing to explore the DWP-CRIS data will first need to submit a project proposal to the CRIS Oversight Committee. The CRIS Oversight Committee will assess whether the application adherences to the agreed standards of research and dissemination specifically outlined for the CRIS database. Once approved, the application will be directed to the Work and Health Screening Panel, specifically set up to consider applications to explore the linked DWP-CRIS data. This panel is made up of a representative from the DWP and a member of the CRIS Oversight Committee. The decision to grant or deny approval for the application to access and use the linked data will be informed by the governance and ethical approvals obtained and implemented as part of the established linkage. These include: 1) NHS Health Research Authority Research Ethics Committee approval, 2) Section 251 approval under the NHS Health Research Authority Confidential Advisory Group, 3) SLaM Caldicott Guardian, 4) DWP governance panels and 5) DWP/CRIS data sharing agreement. In addition, all projects are required to have a local collaborator from King's Health Partners (e.g. SLaM, King's College London, King's College Hospital or Guy's and St Thomas' NHS Foundation Trust).

All approved projects are published with the proposal title, lay summary and lead researcher details on the public facing Maudsley BRC website (https://www.maudsleybrc.nihr.ac.uk/facilities/clinical-record-interactive-search-cris/cris-data-linkages). All research papers will be published in the CRIS publications section of the BRC website (https://www.maudsleybrc.nihr.ac.uk/facilities/clinical-record-interactive-search-cris/cris-publications/).

Once the Work and Health Screening Panel has approved the application, the applicant will work with the SLaM Clinical Data Linkage Service to develop a project data extraction specification, only including the data that is needed to answer the specific research questions as outlined in the project application. The analysis of specific extracts of the linked data will be carried out within the SLaM firewall by the applicant on site, or via a secure VPN connection. Only those who hold a contract with SLaM (substantive or honorary), or a research passport, will be able to submit a project application and work with the linked data once approved.

For peer review only

Supplementary Table 1: Overview of types of benefits received among linked patients (n=183,821).

**Type of benefit**µ¬	**N (%)**
Employment and Support Allowance (ESA)	82436 (44.9)
Jobseeker's Allowance (JSA)	75524 (41.1)
Income Support (IS)	59748 (32.5)
Disability Living Allowance (DLA)	52675 (28.7)
Incapacity Benefit (IB)	50520 (27.5)
Retirement / State Pension (RP)	49040 (26.7)
Personal Independence Payment (PIP)	47315 (25.7)
Universal Credit (UC)	46789 (25.4)
UC conditionality regime – Searching for work	38073 (81.4)
UC conditionality regime – Working, with requirements	13448 (28.7)
UC conditionality regime – No work requirements	16505 (35.3)
UC conditionality regime – Working, no requirements	13610 (29.1)
UC conditionality regime – Preparing for work	4497 (9.6)
UC conditionality regime – Planning for work	2402 (5.1)
Attendance Allowance (AA)	25017 (13.6)
Pension Credit (PC)	22749 (12.4)
Carer's Allowance (ICA)	13798 (7.5)
Severe Disablement Allowance (SDA)	3682 (2.0)
Passported Incapacity Benefit (PIB)	1622 (0.9)
Bereavement Benefit (BB)	732 (0.4)
Widows Benefit (WB)	326 (0.2)

µ benefit received between 1st of January 2005 and 30th of June 2020. ¬ PIP was only introduced in April 2013 to replace DLA. UC was only introduced in 2013. SDA was replaced by IB in April 2001. IB was replaced by ESA and since January 2011 no new IB claims have been accepted. % will not add up to 100% as patients could have received multiple benefits over time.

For peer review only

Supplementary Table 2: Overview of patients who had a recorded primary psychiatric diagnosis and had ever a benefit entry for benefits not directly related to unemployment, sickness, disability, Income Support or Universal Credit.

**Benefit type** / **Recorded primary psychiatric diagnoses (ICD-10 code and description) ∞**	**Retirement / State Pension (RP) N (%)** n=22605	**Pension Credit (PC) N (%)** n=18358	**Attendance Allowance (AA) N (%)** n=20870	**Widow's Benefit (WB) N (%)** n=224	**Bereavement Benefit (BB) N (%)** n=502	**Carer's Allowance (ICA) N (%)** n=9298	**Passported Incapacity Benefit (PIB) N (%)** n=1194
F00-F09 (Mental and behavioural disorders, and mental disorders due to known physiological conditions) n=26069	22605 (86.7)	9827 (37.7)	15503 (59.5)	73 (0.3)	44 (0.2)	1146 (4.4)	32 (0.1)
F10-F19 (Mental and behavioural disorders due to psychoactive substance use) n=23713	1879 (7.9)	1118 (4.7)	413 (1.7)	19 (0.1)	68 (0.3)	2002 (8.4)	89 (0.4)
F20-F29 (Schizophrenia, schizotypal, delusional disorders and other non-mood psychotic disorders) n=14944	2732 (18.3)	2042 (13.7)	715 (4.8)	19 (0.1)	39 (0.3)	520 (3.5)	183 (1.2)
F30-F39 (Mood (affective) disorders) n=27046	6502 (24.0)	2996 (11.1)	2532 (9.4)	58 (0.2)	178 (0.7)	2426 (9.0)	122 (0.5)
F40-F48 (Anxiety, dissociative, stress-related, somatoform and other nonpsychotic mental disorders) n=20432	4128 (20.2)	1765 (8.6)	1567 (7.7)	46 (0.2)	134 (0.7)	1787 (8.8)	197 (1.0)

For peer review only

F50-F59 (Behavioural syndromes associated with physiological disturbances and physical factors) n=3840	226 (5.9)	64 (1.7)	40 (1.0)	<5 (<1.0)	18 (0.5)	276 (7.2)	50 (1.3)
F60-F69 (Disorders of adult personality and behaviour) n=5495	316 (5.8)	205 (3.7)	64 (1.2)	<5 (<1.0)	12 (0.2)	437 (8.0)	77 (1.4)
F70-F79 (Intellectual disabilities) n=2448	233 (9.5)	299 (12.2)	26 (1.1)	<5 (<1.0)	<5 (<1.0)	41 (1.7)	232 (9.5)
F80-F89 (Pervasive and specific developmental disorders) n=2623	39 (1.5)	20 (0.8)	5 (0.2)	<5 (<1.0)	<5 (<1.0)	145 (5.5)	116 (4.4)
F90-F98 (Behavioural and emotional disorders with onset usually occurring in childhood and adolescence) n-5092	59 (1.2)	22 (0.4)	5 (0.1)	<5(<1.0)	6 (0.1)	518 (10.2)	96 (1.9)

∞ latest psychiatric primary diagnosis recorded closest and before window end date (30 June 2019) based on ICD-10 F codes only (mental and behavioural disorders) but excluding non-specific diagnoses, e.g. Z, F99*, FXX. µ any type of benefits received between 1st of January 2005 and 30th of June 2020.* Cell sizes with less than <5 observations are shown as <5 (<1.0%).

For peer review only

Supplementary Table 3: Overview of patients who had a recorded primary psychiatric diagnosis and had received Universal Credit, by Universal Credit conditionality regime.

**Benefit type** / **Recorded primary psychiatric diagnoses (ICD-10 code and description) ∞**	**UC Conditionality regime – searching for work (AA)** **N (%)** n=25012	**UC Conditionality regime – working, with requirements (AB)** **N (%)** n=8409	**UC Conditionality regime – no work requirements (BC)** **N (%)** n=11404	**UC Conditionality regime – working, no requirements (BD)** **N (%)** n=8450	**UC Conditionality regime – preparing for work (CE)** **N (%)** n=2991	**UC Conditionality regime – planning for work (DF)** **N (%)** n=1488
F00-F09 (Mental and behavioural disorders, and mental disorders due to known physiological conditions) n=513	415 (80.9)	129 (25.2)	240 (46.8)	117 (22.8)	36 (7.0)	6 (1.2)
F10-F19 (Mental and behavioural disorders due to psychoactive substance use) n=8547	7605 (88.7)	1911 (22.3)	2524 (29.4)	1809 (21.1)	807 (9.4)	185 (2.2)
F20-F29 (Schizophrenia, schizotypal, delusional disorders and other non-mood psychotic disorders) n=2989	2467 (85.1)	762 (26.3)	1427 (49.2)	638 (22.0)	113 (3.9)	52 (1.8)
F30-F39 (Mood (affective) disorders) n=7044	5437 (77.2)	2212 (31.4)	2814 (40.0)	2322 (33.0)	866 (12.3)	553 (7.9)
F40-F48 (Anxiety, dissociative, stress-related, somatoform and	4197 (77.0)	1744 (32.0)	2003 (36.8)	1805 (33.1)	650 (11.9)	364 (6.7)

For peer review only

other nonpsychotic mental disorders) n=5451						
F50-F59 (Behavioural syndromes associated with physiological disturbances and physical factors) n=1168	831 (71.2)	332 (28.4)	346 (29.6)	484 (41.4)	110 (9.4)	95 (8.1)
F60-F69 (Disorders of adult personality and behaviour) n=1874	1500 (80.0)	448 (26.0)	934 (49.8)	494 (26.4)	180 (9.6)	94 (5.0)
F70-F79 (Intellectual disabilities) n=238	195 (81.9)	32 (13.5)	143 (60.1)	18 (7.6)	20 (8.4)	5 (2.1)
F80-F89 (Pervasive and specific developmental disorders) n=653	551 (84.4)	158 (24.2)	285 (43.6)	111 (17.0)	53 (8.1)	17 (2.6)
F90-F98 (Behavioural and emotional disorders with onset usually occurring in childhood and adolescence) n=2209	1814 (82.1)	641 (29.0)	688 (31.2)	652 (29.5)	156 (7.1)	117 (5.3)

∞ latest psychiatric primary diagnosis recorded closest and before window end date (30 June 2019) based on ICD 10 F codes only (mental and behavioural disorders) but excluding non-specific diagnoses, e.g. Z, F99*, FXX. μ any type of benefits received between 1st of January 2005 and 30th of June 2020.*

For peer review only
